# Global, regional, and national burden of fracture of sternum and/or fracture of one or more ribs: a systematic analysis of incidence, YLDs with projections to 2030

**DOI:** 10.3389/fpubh.2025.1565478

**Published:** 2025-04-03

**Authors:** Zhizhi Wang, Yikuan Cai, Yu Tong, Huajie Li, Hu Zhou, Tongyin Ou, Tianlan Ye, Jiangsheng Zhang, Kaican Cai, Zhiming Chen

**Affiliations:** ^1^Department of Thoracic Surgery, Nanfang Hospital, Southern Medical University, Guangzhou, China; ^2^The Second School of Clinical Medicine, Southern Medical University, Guangzhou, China; ^3^The First School of Clinical Medicine, Southern Medical University, Guangzhou, China; ^4^School of Public Health, Southern Medical University, Guangzhou, China

**Keywords:** rib fracture, sternal fracture, global burden of disease (GBD), BAPC model, trends

## Abstract

**Background:**

Sternal and/or rib fractures represent a growing global health challenge. Despite their significant clinical and public health implications, epidemiological studies on these injuries remain limited. Utilizing the Global Burden of Disease 2019 (GBD2019) database, this study evaluates the worldwide burden of sternal and/or rib fractures and projects trends through 2030 to inform policy development.

**Methods:**

We analyzed global incidence, age-standardized incidence rate (ASIR), years lived with disability (YLDs), age-standardized YLD rate (ASYR) and estimated annual percentage change (EAPC) of sternal and/or rib fractures across geographic regions, nations, age-sex groups, and socio-demographic index (SDI) quintiles using GBD2019 data. The Bayesian age-period-cohort (BAPC) model was employed to forecast trends until 2030.

**Results:**

In 2019, global incident cases of sternal/rib fractures reached 4.1 million (44% increase since 1990), with YLDs at 190,000 (62% rise since 1990). While ASIR and ASYR showed modest declines, the absolute burden remained substantial. East Asia and high-income North America both exhibited the highest incident cases and YLDs. Central Latin America and Western Saharan Africa demonstrated the steepest incidence growth, whereas the Caribbean experienced the most pronounced YLD increases. Nationally, China reported the highest absolute burden, while Greenland had the highest ASIR and ASYR. Males carried over 60% of the burden. Also, there was a negative correlation between EAPC and national SDI. Projections indicate that the global burden of disease will continue to rise by 2030.

**Conclusion:**

The escalating burden of sternal/rib fractures demands targeted interventions. Prioritizing injury prevention in high-burden regions (e.g., East Asia), addressing male-dominated occupational/behavioral risks, and optimizing infrastructure such as medical transport in low-SDI settings are critical policy priorities. Sustained surveillance through standardized reporting systems is essential for monitoring progress.

## Introduction

Fractures have become a greater health problem worldwide (1), especially in patients with osteoporosis, which places a heavy burden on the world (2, 3). Sternal and rib fractures are categorized in the same category due to their similar anatomical and therapeutic relationship. The location of sternal fractures can be categorized into sternal styloid, sternal body, and raphe (4). Rib fractures can be categorized into single and multiple rib fractures based on the number of broken ends.

Sternal and/or rib fractures are a common type of fracture with high morbidity and mortality (5). Common causes of sternal and/or rib fractures are traffic accidents, falls and crush injuries (6–9). The Agency for Healthcare Research and Quality (AHRQ) reported a 19.4% increase in emergency department visits for rib fractures in the United States from 2006 to 2014 (5). In the Netherlands, 37% of patients with rib fractures were admitted to the ICU (10). The total direct cost of osteoporosis in Australia in 2017 was estimated to be a $3.44 billion, with fracture treatment accounting for 68% of the total cost (11).

Minor rib fractures heal on their own, but multiple rib fractures significantly increase the incidence of complications such as pneumonia, acute respiratory distress syndrome (ARDS), and pneumothorax (12). The incidence of complications and subsequent mortality is higher in older adult patients (13, 14). Surgical treatment of sternal and/or rib fractures with optional surgical fixation improves survival after major thoracic trauma (15, 16) and improves prognosis (17). Non-surgical treatments are equally important, such as oral analgesics. However, the indications for specific treatment are not standardized. The thoracic cavity contains important structures, and sternal and/or rib fractures may result in serious adverse outcomes (18) or even life-threatening (19). The burden of sternal and/or rib fractures is rising and there is a gap in prevention and treatment (16), so it is important to analyze and summarize the epidemiological characteristics of sternal and/or rib fractures, which are important to reduce the burden of fractures.

The GBD2019 database provides a global research platform, documenting 369 diseases and injuries in 204 countries and territories (20). In this cross-sectional study, we used the GBD2019 database to systematically analyze and summarize the age-standardized incidence rate (ASIR) and age-standardized YLD rate (ASYR) of sternal and/or rib fractures based on multiple dimensions of region, country, age, sex, and SDI. Through epidemiological analyses of different regions and populations, we provided detailed epidemiological information, which revealed the differences in the burden of sternal and/or rib fractures globally today. We adopted the Bayesian Age-Period-Cohort (BAPC) model, taking advantage of its ability to separate the risk at specific ages from temporal trends, and visualized the credible intervals to generate robust predictions up to 2030. This approach demonstrates superior predictive accuracy compared to conventional modeling approaches, thereby equipping policymakers with temporally stratified intervention windows to optimize resource allocation strategies, to reduce the global burden of sternal and/or rib fractures.

## Methods

### Data source

This analysis of the burden of sternal fracture and/or one or more rib fractures is based on the database of the Global Burden of Disease Study 2019 (GBD 2019). The database encompasses incidence and years lived with disability (YLDs), for 369 distinct diseases and injuries across 204 countries and territories (20, 21). GBD 2019 draws upon a multitude of data sources, including household surveys, disease registries, healthcare utilization records, and population censuses (20, 22).

### Definitions

In the GBD 2019, injuries are classified according to two dimensions: cause and nature. The causes of injury are exemplified by traffic accidents and falls, and so on. In contrast, the nature of injury pertains to the physical consequences of these causes, such as rib fractures (23). YLDs are used to measure non-fatal health losses due to disease in populations. The SDI is a composite measure of a country or region’s level of social and demographic development. It takes values ranging from 0 to 1 and is used to categorize regions and countries into five groups: high, medium-high, medium, medium-low, and low (24, 25).

The ASIR and ASYR are two standardized rate indicators used in epidemiology. The ASIR is used to correct for the effect of age composition on morbidity by providing standardized rates of new cases that are comparable across populations. In contrast, the ASYR measures the loss of health due to disease or injury, taking into account the age structure of the population. The EAPC is expressed as the average annual percentage change in each health indicator over a fixed time interval, thereby revealing the changing dynamics of health trends.

### Data analysis

The incidence rate, ASIR, and ASYR associated with sternal and/or ribs fractures between 1990 and 2019 were calculated for the metrics and expressed as 95% uncertainty intervals (95% UI). Furthermore, we explored the disease-related incidence, ASIR, and ASYR from different dimensions, including region, age, gender, and SDI. We calibrated the population per 100,000 and considered the age distribution to calculate the age-standardized rate (ASR) and calculated the EAPC based on the ASR data for each year of the GBD. The computation of the EAPC was based on the assumptions of a log-linear model, which is expressed by the following equation: y = *α* + β*x + *ϵ*, with y representing ln (ASRs), x representing year, and β representing the regression coefficient. The EAPC was calculated using the formula $\mathrm{EAPC}=100*\left(e^{\beta }-1\right)$ , and the 95% confidence intervals (95% CI) for the EAPC were obtained through a linear regression model (26). When both the EAPC and the lower 95% CI are greater than zero, this indicates a positive trend in the change of the ASR, which may be understood as an increase over time. Conversely, when both the EAPC and the upper 95% CI of the ASR are less than zero, this suggests a negative trend in the change of the ASR, which may be understood as a decrease over time. The remaining results were deemed to represent statistically insignificant changes in ASR.

Bayesian age-period-cohort (BAPC) analysis was conducted using the GBD2019 database to predict the global burden of disease for rib fractures between 2020 and 2030. Specifically, we collated and analyzed global and regional data on the incidence and YLDs of rib fractures between 1990 and 2019, grouped by 5-year age cohorts. Finally, we employed the BAPC and INLA packages in the R software for model fitting and prediction (27). All analyses and visualizations were conducted using the R statistical software (version 4.2.3).

## Results

### Global trends

In 2019, the global incidence number of new cases of sternal and/or rib fractures was 4,109,551 (95% UI: 2970312 to 5,820,023) with an ASIR of 52.22 (95% UI: 37.86 to 74.18) per 100,000 people. The number of incidence increased by about 44% compared to 1990 (incidence number: 2860073, 95% UI: 2077796 to 3,974,637). In terms of YLDs, in 2019, the YLDs for sternal and/or rib fractures were 190,834 (95% UI: 127739 to 272,079) with an ASYR of 2.37 (95% UI: 1.58 to 3.37). From 1990–2019, the number of YLDs increased from 117,528 (95% UI: 78613 to 168,926) to 190,834 (95% UI: 127739 to 272,079), a 62% increase. Encouragingly, the ASIR and ASYR of sternal and/or rib fractures showed a slight decreasing trend in the last three decades, with an EAPC of −0.34 (95% CI: −0.46 to −0.22) and − 0.42 (95% CI: −0.53 to −0.31), respectively (Supplementary Table 1).

### Regional and national trends

**Figure 1:**
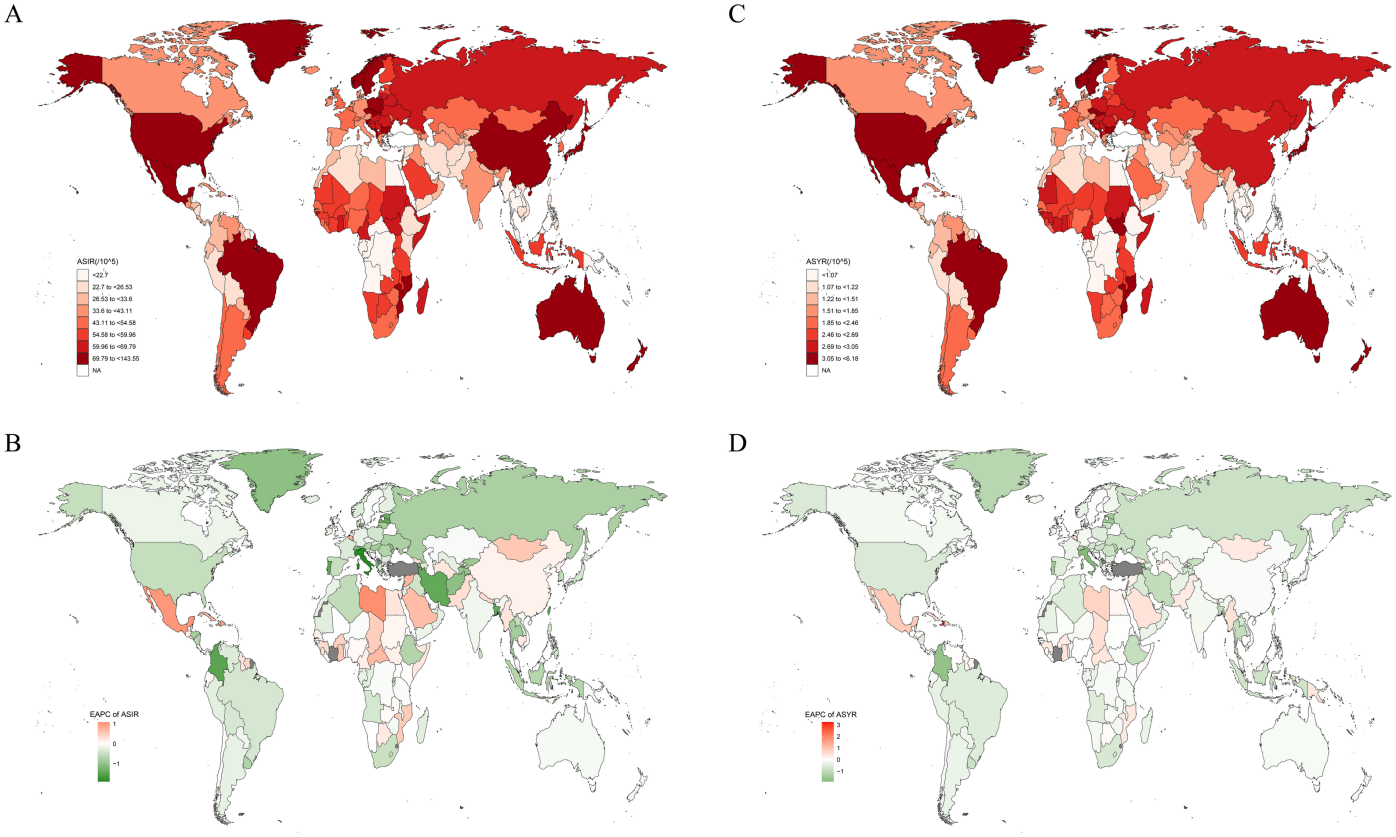
The spatial distribution of sternal and/or ribs fractures ASIR **(A)** and ASYR **(C)** in 2019, and the EAPC of ASIR **(B)** and ASYR **(D)**. ASIR, age-standardized incidence rate; ASYR, age-standardized YLD rate; EAPC, estimated annual percentage change.

**Figure 2:**
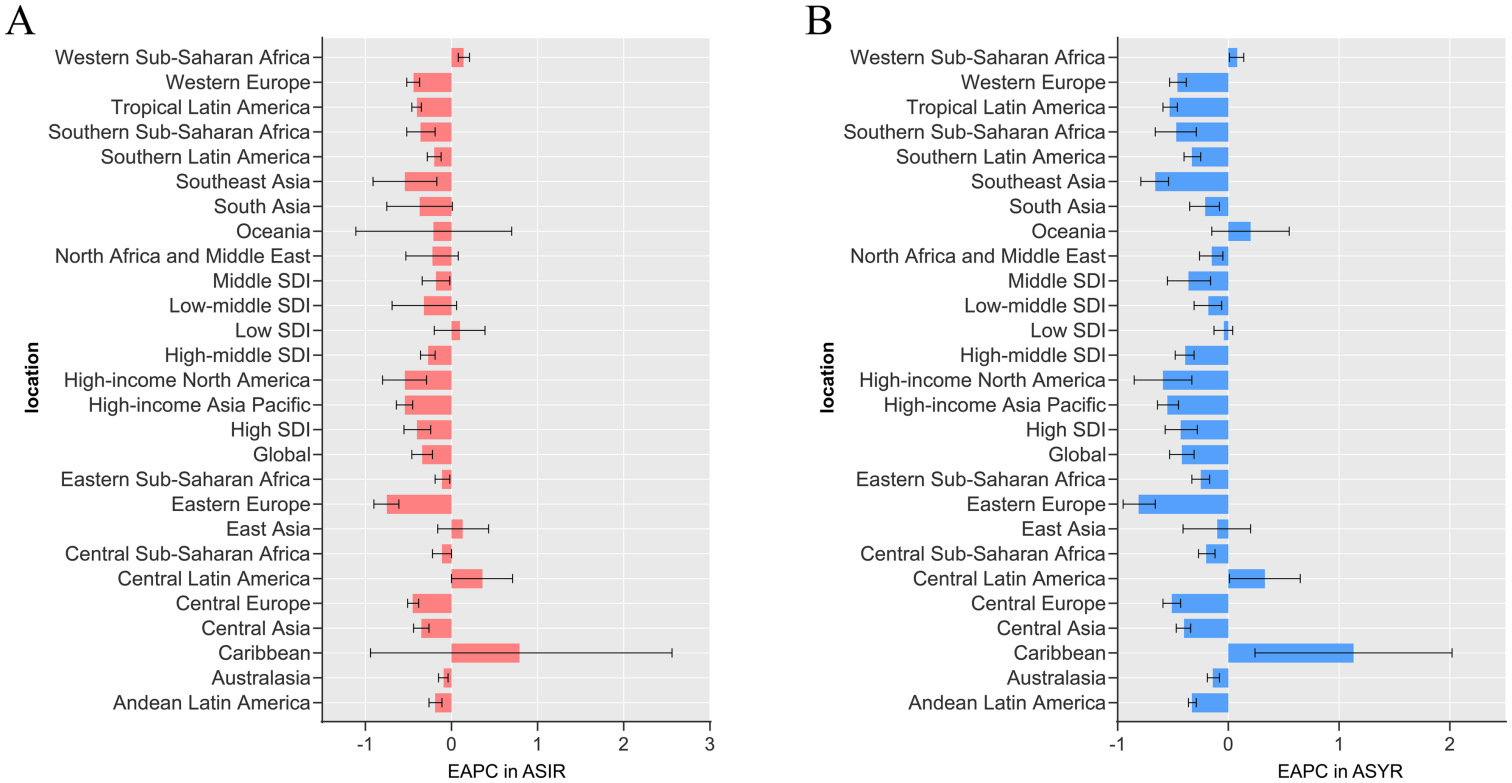
Estimated annual percentage change (EAPC) in ASIR **(A)** and ASYR **(B)** of sternal and/or ribs fractures in 2019 in 26 GBD regions. Error bars indicate the 95% UI for the EAPC. ASIR, age-standardized incidence rate; ASYR, age-standardized YLD rate; UI, uncertainty interval.

**Figure 3:**
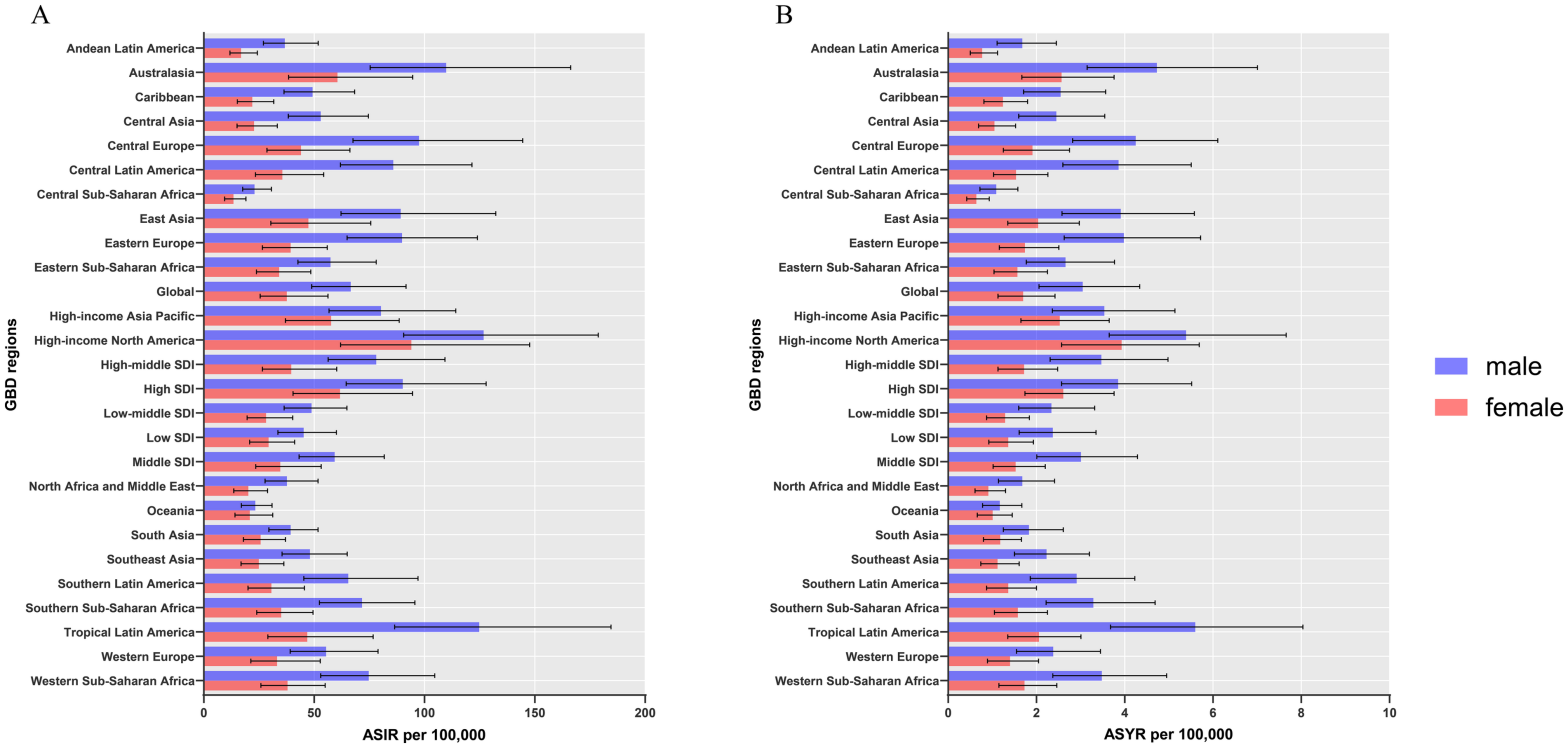
ASIR **(A)** and ASYR **(B)** of sternal and/or ribs fractures by sex, across 26 GBD regions, in 2019. Error bars indicate the 95% UI for the ASR. ASIR, age-standardized incidence rate; ASYR, age-standardized YLD rate; UI, uncertainty interval; ASR, age-standardized rates.

The region with the highest burden of sternal and/or rib fractures in 1990 and 2019 was East Asia (Figure 1), reporting the highest number of incidence, YLDs, which were 1,159,376 (95% UI: 782424 to 1,732,608), and 55,778 (95% UI: 36863 to 78,918) in 2019, respectively. In 2019, the highest ASIR and ASYR in the world were both in High-income North America (Supplementary Table 1). Among all GBD regions, only Central Latin America and Western Sub-Saharan Africa regions showed statistically significant increasing trends in ASIR. The increasing trend was more pronounced in Central Latin America (EAPC: 0.36, 95% CI: 0 to 0.71) (Supplementary Table 1 and Figure 2A). The most significant increasing trend in ASYR was observed in the Caribbean region (EAPC: 1.13, 95% CI: 0.24 to 2.02), which contrasts with most of the regions that showed a decreasing trend in ASYR. Meanwhile, Central Latin America and Western Sub-Saharan Africa regions also showed an increasing trend in ASYR (Supplementary Table 1 and Figure 2B). At the gender level, the burden is significantly greater for males than for females in all regions of the globe (Figure 3).

During the same period, China had the highest incidence number, YLDs globally, and Greenland had the highest ASIR, ASYR. Libya had the highest increase in ASIR with an EAPC of 1.08 (95% CI: 0.39 to 1.79); and Haiti had the highest increase in ASYR with an EAPC of 3.23 (95% CI: 1.36 to 5.14). Implying that these two countries had the fastest growing sternal and/or rib fractures burden. Notably, the country with the most significant downward trend in ASIR and ASYR was Italy, with an EAPC of −1.9 (95% CI: −2.29 to −1.51) and −1.89 (95% CI: −2.29 to −1.49), respectively (Supplementary Table 2). ASIR and ASYR showed a decreasing trend in most countries (Figure 1).

### Global trends by SDI

**Figure 4:**
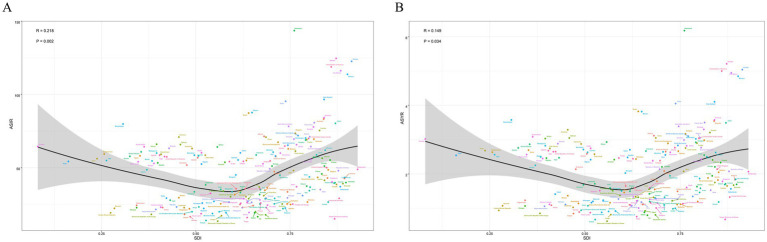
Correlation between SDI and sternal and/or ribs fractures ASIR **(A)**, ASYR **(B)** across 204 countries in 2019. SDI, sociodemographic index; ASIR, age-standardized incidence rate; ASYR, age-standardized YLD rate.

**Figure 5:**
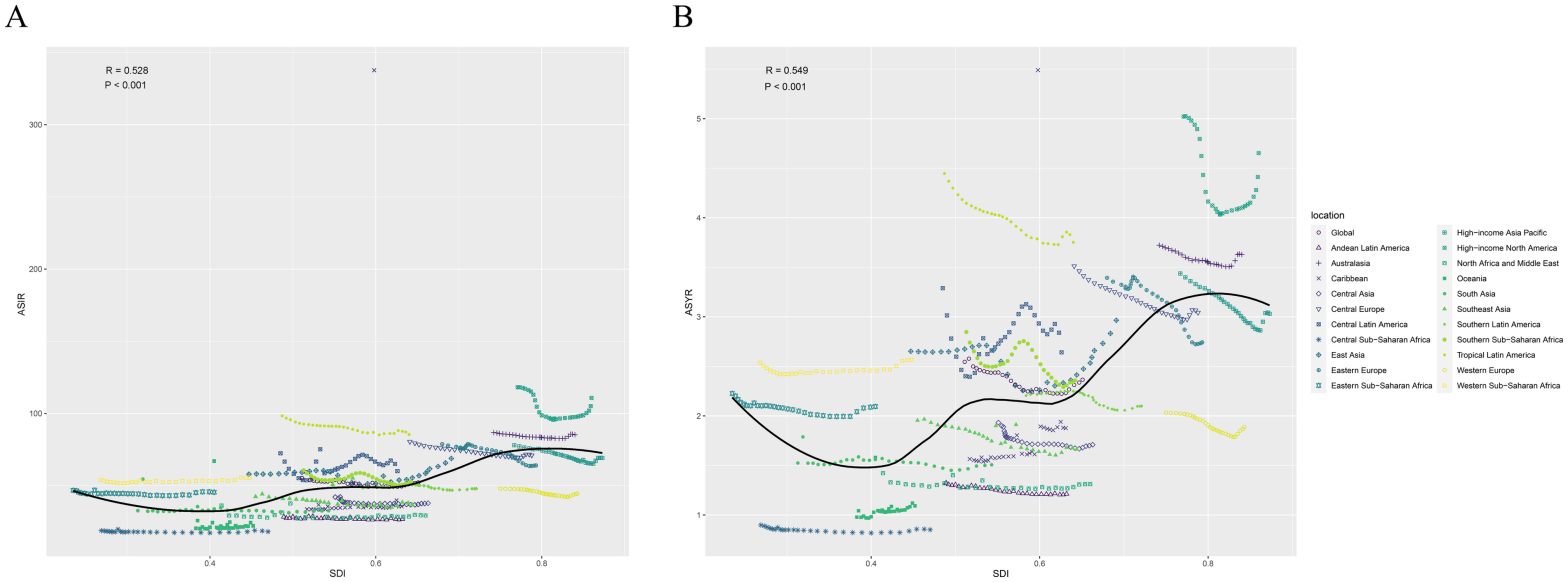
Correlation between SDI and sternal and/or ribs fractures ASIR **(A)**, ASYR **(B)** across 26 GBD regions in 2019. ASIR, age-standardized incidence rate; ASYR, age-standardized YLD rate; SDI, sociodemographic index.

There is a “v” shaped correlation between ASIR, ASYR and SDI across countries in 2019. As the SDI of different countries increases, ASIR and ASYR first decrease and then increase, with an inflection point around 0.625. Among them, the ASIR and ASYR of Greenland are significantly higher than those of countries with the same SDI, and Greenland also has the highest ASIR and ASYR in the world (Figure 4). Looking at the SDI of GBD regions, both ASIR (*R* = 0.528, *p* < 0.001) and ASYR (*R* = 0.549, *p* < 0.001) show an upward trend as the SDI increases. Among them, the change of ASIR and ASYR with the fluctuation of SDI is more obvious in High-income North America, which is also the region with the highest ASIR and ASYR in the world (Figure 5).

**Figure 6:**
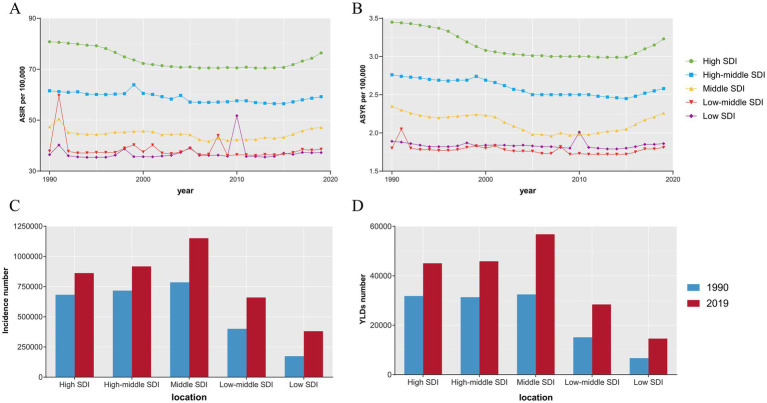
The ASIR **(A)**, ASYR **(B)** and incidence number **(C)**, YLDs number **(D)** of sternal and/or ribs fractures in different SDI regions. ASIR, age-standardized incidence rate; ASYR, age-standardized YLD rate; YLDs, years lived with disability; SDI, sociodemographic index.

In the last three decades, the ASIR and ASYR of the High SDI and High-middle SDI regions have been consistently high; the ASIR and ASYR of the Low SDI and Low-middle SDI regions have been at a lower level globally and have fluctuated more (Figures 6A,B). In 1990, High SDI, High-middle SDI, and Middle SDI regions had relatively similar incidence counts and YLDs; however, in 2019, Middle SDI regions reported the highest incidence number and YLDs globally. In addition, the largest increase in incidence number and YLDs was in Low SDI regions, with an increase of 119%, which is significantly higher than other SDI regions (Figures 6C,D and Supplementary Table 1).

### Global trends by age and sex

**Figure 7:**
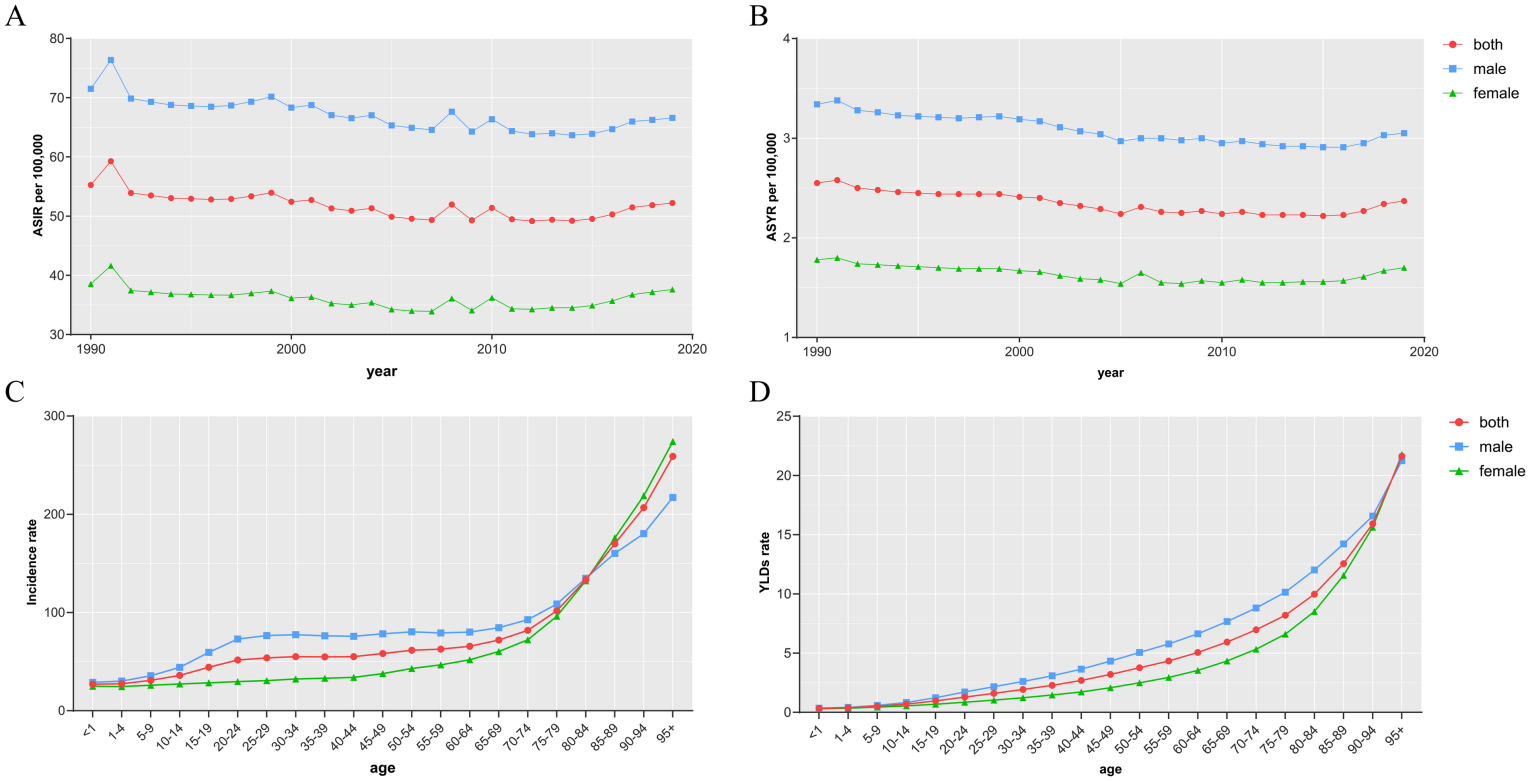
The ASIR **(A)** and ASYR **(B)** of sternal and/or ribs fractures by sex from 1990 to 2019, and the age-specific numbers of incidence **(C)** and YLDs **(D)** in 2019. ASIR, age-standardized incidence rate; ASYR, age-standardized YLD rate; YLDs, years lived with disability.

Globally, the ASIR and ASYR for females in 2019 were 37.6 and 1.7 per 100,000, respectively, compared to 66.58 and 3.05 for males. The gap between males and females persisted in 1990–2019 and the burden for males has been significantly higher than that for females. ASIR and ASYR fluctuated within a range and showed a downward trend for both males and females during the three decades. The trends are similar for both (Figures 7A,B). After dividing the population according to age, the number of morbidities has been consistently higher in males than in females in the population up to the age of 85 years. However, after the age of 85, the number of incidence in females exceeded that of males (Figure 7C). In the total population, YLDs gradually increased with age in both sexes. YLDs were consistently higher in males than in females up to the age of 95 years, and were almost identical in both sexes in the population over the age of 95 years (Figure 7D).

### Correlations of EAPC with SDI

**Figure 8:**
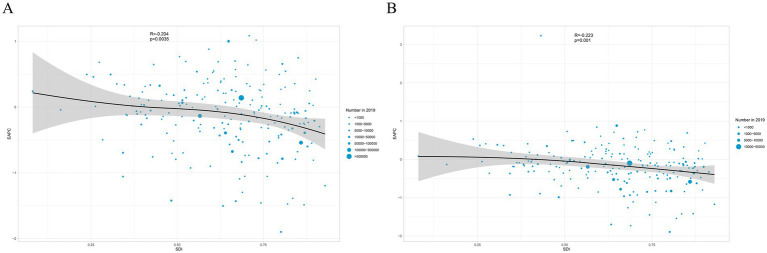
Correlation between SDI and EAPC in ASIR **(A)** and ASYR **(B)** of sternal and/or ribs fractures in 2019. The circles represent countries that were available on SDI data. The size of circles indicates the number of spinal cord injury patients in 2019. SDI, sociodemographic index; ASIR, age-standardized incidence rate; ASYR, age-standardized YLD rate.

After calculating the EAPCs of different countries, we found that there was a statistically significant correlation between the EAPCs of each of the different countries and the SDI of that country. There was a negative correlation between the EAPC of ASIR and the SDI (*R* = −0.204, *p* = 0.0035). The EAPC of ASYR also showed a negative correlation with the SDI (*R* = −0.223, *p* = 0.001) (Figure 8).

### Projections up to 2030

**Figure 9:**
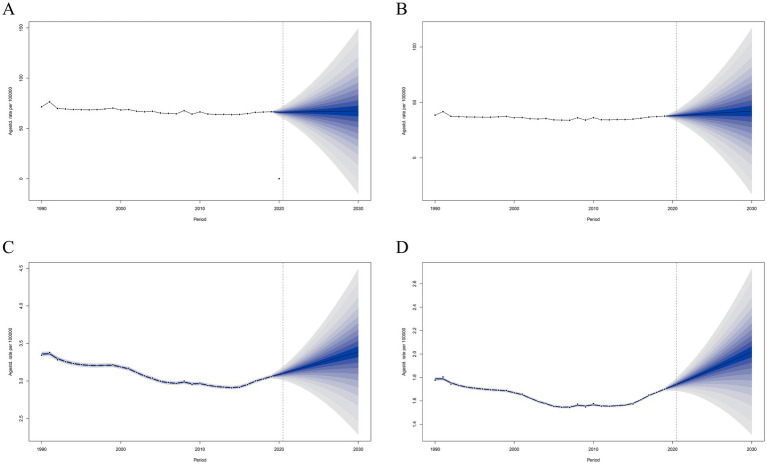
Projections of ASIR **(A,B)** and ASYR **(C,D)** in males and females from 2020 to 2030. The open dot represents the observed value, and the fan shape, the predicted distribution between the 2.5 and 97.5% quantiles. The average forecast is shown as a solid line. The vertical dotted line indicates where the prediction begins. ASIR, age-standardized incidence rate; ASYR, age-standardized YLD rate.

**Figure 10:**
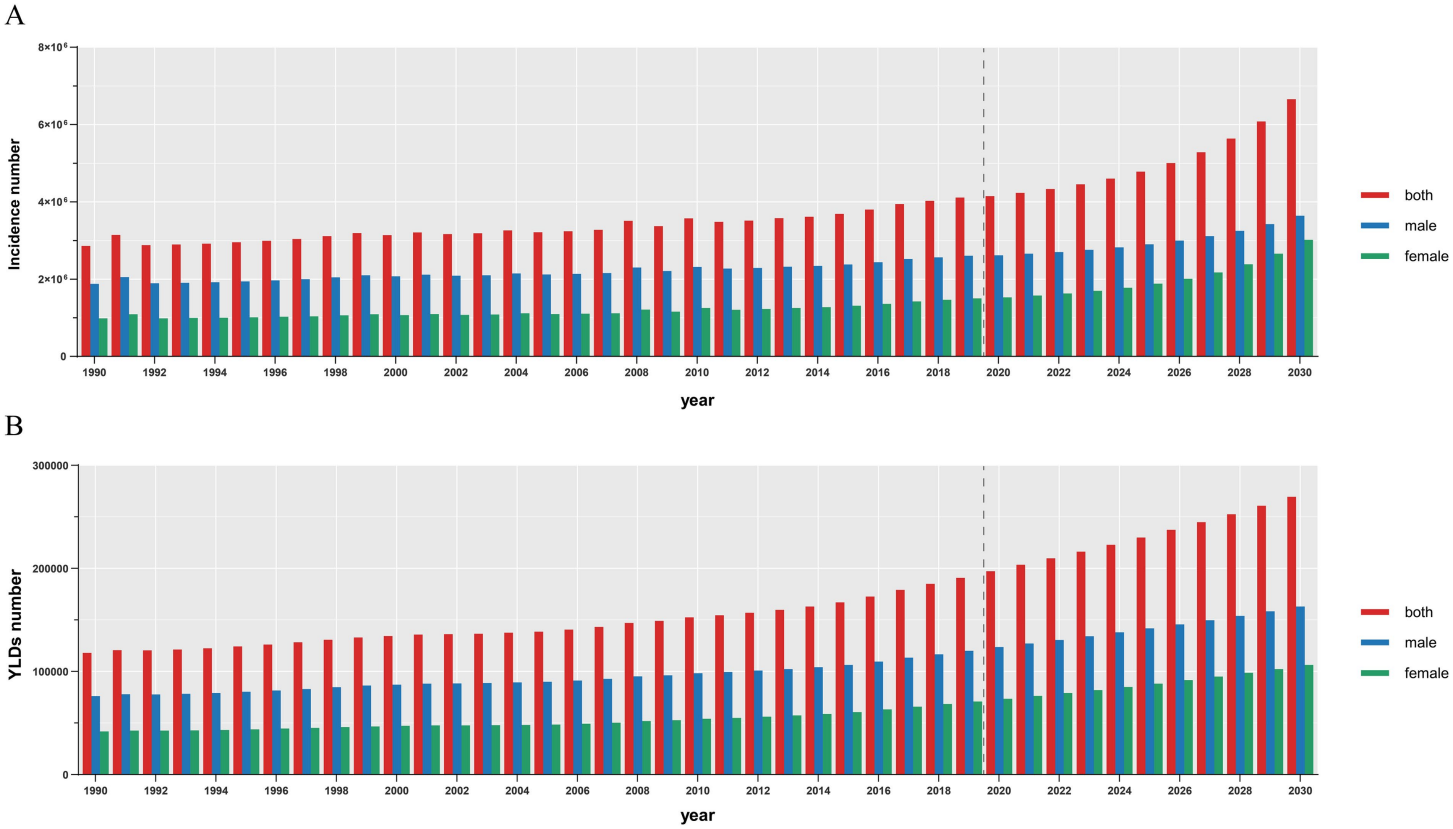
Projections number of ASIR **(A)** and ASYR **(B)** in both sexes from 2020 to 2030. The vertical dotted line indicates where the prediction begins. ASIR, age-standardized incidence rate; ASYR, age-standardized YLD rate. YLDs, years lived with disability.

By 2030, the number of incidence of sternal and/or rib fractures and YLDs of sternal and/or rib fractures will continue to rise globally (Figure 9). According to our projections, the number of incidence cases will increase by more than 60% and YLDs will increase by 40% in both sexes in the 11-year period from 2019 to 2030. In addition, the number of incidence will increase more in females than in males, implying that the gap between the sexes will gradually narrow (Figure 10). Overall, the burden of sternal and/or rib fractures will continue to rise in the future, and will be higher in males than in females over the next decade.

## Discussion

This study used the GBD2019 database to systematically analyze and summarize the epidemiological characteristics of fracture of sternal and/or rib fractures worldwide. This is the first study in the world to measure and predict the burden of sternum and/or rib fractures in multiple dimensions such as number of incidence, YLDs, region, and age. Our findings emphasize the global burden of sternal and/or rib fractures and the impact on individuals and society. Supplementary Tables 1, 2 present the incidence rates of sternum/rib fractures, data on disability-adjusted life years (YLDs), and annual trends (EAPCs) of sternum/rib fractures in 26 GBD regions and 204 countries worldwide from 1990 to 2019, respectively, which can be used as a reference.

The results show that over the last three decades, although ASIR and ASYR have shown a slight downward trend. But the numbers are worrisome, with a 44% increase in the number of sternal and/or rib fractures and a 62% increase in YLDs in 2019 compared to 1990. This phenomenon can be attributed to the aging of the world population (28) and the increase in the total population base. Over the past three decades, the world population has increased by an average of 83.8 million people per year, from 5.3 billion in 1990 to 7.7 billion in 2019, an increase of 45% (29). YLDs for sternal and/or rib fractures were higher in males than females at all ages throughout the study period, although there was a convergence between the sexes in the 95+ age group.

In terms of the spatial distribution of the burden of sternal and/or rib fractures, the burden of sternal and/or rib fractures varies considerably across regions and countries. In terms of incidence and YLDs, the highest burden is in East Asia, which can be attributed to the large population of East Asia, which has 22% of the world’s population but 28% of the global burden of sternal and/or rib fractures. In terms of ASIR and ASYR, the highest burden of ASIR and ASYR globally is found in High-income North America. Meanwhile, ASIR and ASYR show an upward trend with the growth of SDI. In more economically developed regions, frequent traffic accidents and trauma following high-energy activities lead to a higher incidence of sternal and/or rib fractures (30). On the other hand, sound medical care and efficient transportation systems, thereby elevating the number of incidence of sternal rib fractures and YLDs in more economically developed regions. The ASIR, ASYR in Low SDI and Low-middle SDI regions were consistently at the lower levels globally and fluctuated considerably during the study period. This may be limited by poorer local healthcare and public services, missing some sternal rib fracture patients and unstable data reporting.

China and India reported the highest number of sternal and/or rib fractures incidence and YLDs globally, with China having more than twice as many as India. This can be attributed to the huge population size of China and India. Meanwhile, globally, Greenland had the highest ASIR and ASYR. This may be related to Greenland’s long, ice-covered winters and the higher risk of traumatic injuries, such as falls and impacts, among residents during their daily activities (e.g., fishing, snow transport). One Study have shown that a lower percentage of patients are treated for osteoporosis in Greenland compared to Denmark (31). Another case report suggests that Greenland has poorer infrastructure and difficulties in transferring patients (32). These may have contributed to Greenland’s higher ASIR and ASYR. Greenland should prioritize interventions to mitigate winter injury risks by equipping high-risk occupational groups with anti-slip gear and safety training, enhancing osteoporosis management to reduce fragility fractures, and modernizing emergency medical transportation systems (such as the rapid transfer of critically ill patients by air). These strategies align with the WHO’s injury prevention framework. It is noteworthy that Libya has the fastest growing ASIR and ASYR in Haiti. Over the twentieth century, Libya and Haiti have seen a dramatic increase in the ASIR and ASYR due to frequent natural disasters, violent conflicts and sharp deterioration of health systems, health issues have faced major challenges (33, 34). In socially unstable countries, not only the burden of bone fractures is high, but Libya also faces a greater burden in terms of mental health, such as post-traumatic stress disorder (PTSD) and depression (35).

In terms of age and sex incidence, the number of sternal and/or rib fractures is significantly higher in males than females up to the age of 84 years. The higher fracture burden among males likely stems from occupational and behavioral risks. Males are disproportionately exposed to physical hazards in sectors like construction and transportation, and exhibit greater risk-taking behaviors (e.g., contact sports, alcohol use) linked to traumatic injuries. Conversely, while females more frequently engage in domestic tasks (lower-energy activities) (36). The number of sternal and/or rib fractures in women starts to increase rapidly after the age of 65 years and exceeds the number of sternal and/or rib fractures in men after the age of 85 years. This is associated with an increased prevalence of osteoporosis in postmenopausal women, which increases the ASIR and ASYR of sternal and/or rib fractures (37). Postmenopausal women are more prone to osteoporotic fractures as they lose the protection of estrogen and have lower bone mass and bone strength (38). The pattern of change is similar for other fracture types (39). A Danish study in middle-aged and older adults showed that the ratio of major osteoporotic fractures in men and women was 1:2–3 (40), which is consistent with our findings. Therefore, screening, prevention, and treatment of osteoporosis in middle-aged and older adults, especially postmenopausal women, are crucial to reduce the burden of fracture disease (38, 41). Middle-aged and older women should be proactive and pay early attention to their bone density and osteoporosis fractures, and take appropriate nutrient supplements when necessary to prevent estrogen deficiency. The healthcare system should also be proactive in identifying early screening markers for osteoporosis to address the problem earlier (42).

The EAPC was negatively correlated with SDI in both ASIR and ASYR, respectively. This suggests that countries with a lower SDI have a higher EAPC, and that the burden of sternal and/or rib fractures in these countries will increase faster than in countries with a higher SDI. In addition, this study used the BAPC method to predict the number of incidence and YLDs in 2030 for both sexes, respectively. This suggests that the burden of sternal rib fractures may continue to increase in the future and that we should not let our guard down. Several countries in Europe have established major trauma systems (9, 43), which allow for more efficient treatment of traumatized patients and improved survival of traumatized patients.

In this study, we systematically analyzed and summarized the burden of sternal and/or rib fractures in different populations in different regions worldwide by analyzing the GBD2019 database. However, our study still has some shortcomings and limitations. The quality of the data varies considerably across regions, with some areas exhibiting a greater degree of bias due to the inability of patients to access healthcare services. The long-time span of this study renders it susceptible to the influence of advancing diagnostic standards and medical technology over recent decades. Patients may have multiple injury outcomes, and the GBD data only report isolated sternal and/or rib fractures, which cannot be clearly distinguished for multiple injuries. While our uncertainty intervals reflect stochastic and structural variance within the GBD framework, scenario-based sensitivity analyses could further contextualize these estimates. Such explorations, while beyond current GBD protocols, represent valuable extensions for future research.

## Conclusion

Our study provides the status and future projections of the burden of sternal and/or rib fractures. Considering the rising global burden of sternal and/or rib fractures, the development of health policy is of paramount importance. Future efforts should focus not only on preventing sternal and/or rib fractures to reduce the global incidence of sternal and/or rib fractures, but also on treating sternal and/or rib fractures as soon as possible after they occur to improve the quality of life of the patients after treatment and to reduce YLDs. Therefore, governments, healthcare organizations, and individuals should work together, and our ultimate goal is to reduce the burden of sternal and/or rib fractures.

## Data Availability

Publicly available datasets were analyzed in this study. This data can be found: https://www.healthdata.org/data-tools-practices.
