# Supplementary material for: Global, regional, and national burden of fracture of sternum and/or fracture of one or more ribs: a systematic analysis of incidence, YLDs with projections to 2030
Source: Front Public Health. 2025 Apr 3;13:1565478. doi: 10.3389/fpubh.2025.1565478 (PMC12003264; doi:10.3389/fpubh.2025.1565478)
Supplement: Supplementary file 1 [file Table_1.docx]

| Table1. The incidence and YLDs of sternum/ribs fractures in 1990 and 2019 and estimated annual percentage change from 1990 to 2019 across 26 GBD regions. | | | | | | | | | | | | |
| --- | --- | --- | --- | --- | --- | --- | --- | --- | --- | --- | --- | --- |
|  | incidence | | | | | | YLDs | | | | | |
| location | Num_1990 (95% UI) | ASR_1990 (95% UI) | Num_2019 (95% UI) | ASR_2019 (95% UI) | Num_change (95% CI) | EAPC (95% CI) | Num_1990 (95% UI) | ASR_1990 (95% UI) | Num_2019 (95% UI) | ASR_2019 (95% UI) | Num_change (95% CI) | EAPC (95% CI) |
| Global | 2860073 | 55.27 | 4109551 | 52.22 | 0.44 | -0.34% | 117528 | 2.55 | 190834 | 2.37 | 0.62 | -0.42% |
|  | (2077796 to 3974637) | (40.24 to 77.05) | (2970312 to 5820023) | (37.86 to 74.18) | (0.36-0.53) | (-0.46 to -0.22) | (78613 to 168926) | (1.71 to 3.64) | (127739 to 272079) | (1.58 to 3.37) | (0.58 to 0.67) | (-0.53 to -0.31) |
| Andean Latin America | 11290 | 28.34 | 17173 | 26.8 | 0.52 | -0.19% | 412 | 1.32 | 747 | 1.21 | 0.81 | -0.33% |
|  | (8265 to 15619) | (20.8 to 39.43) | (12371 to 24166) | (19.29 to 37.74) | (0.36-0.68) | (-0.26 to -0.11) | (271 to 600) | (0.88 to 1.92) | (497 to 1080) | (0.81 to 1.75) | (0.62 to 1.05) | (-0.36 to -0.29) |
| Australasia | 17456 | 86.79 | 24164 | 85.27 | 0.38 | -0.09% | 802 | 3.72 | 1279 | 3.63 | 0.6 | -0.14% |
|  | (11730 to 27059) | (58.06 to 133.72) | (16366 to 36320) | (57.55 to 129.24) | (0.3-0.5) | (-0.15 to -0.04) | (534 to 1157) | (2.47 to 5.4) | (861 to 1856) | (2.41 to 5.28) | (0.44 to 0.78) | (-0.19 to -0.08) |
| Caribbean | 11943 | 33.7 | 16909 | 35.57 | 0.42 | 0.79% | 487 | 1.57 | 932 | 1.88 | 0.91 | 1.13% |
|  | (8830 to 16168) | (24.91 to 45.55) | (12242 to 23533) | (25.8 to 49.31) | (0.33-0.51) | (-0.94 to 2.56) | (324 to 710) | (1.05 to 2.26) | (627 to 1299) | (1.26 to 2.61) | (0.68 to 1.4) | (0.24 to 2.02) |
| Central Asia | 29954 | 42.08 | 36127 | 37.85 | 0.21 | -0.35% | 1161 | 1.93 | 1542 | 1.71 | 0.33 | -0.40% |
|  | (21488 to 41826) | (30.21 to 58.8) | (25680 to 51036) | (26.89 to 53.38) | (0.16-0.25) | (-0.44 to -0.26) | (778 to 1675) | (1.29 to 2.77) | (1007 to 2231) | (1.12 to 2.46) | (0.23 to 0.44) | (-0.47 to -0.34) |
| Central Europe | 98845 | 80.44 | 79104 | 71.09 | -0.2 | -0.45% | 4686 | 3.51 | 4499 | 3.04 | -0.04 | -0.51% |
|  | (68401 to 142744) | (55.66 to 116.11) | (54170 to 116899) | (48.9 to 104.99) | (-0.25--0.15) | (-0.51 to -0.38) | (3121 to 6728) | (2.34 to 5.07) | (2979 to 6423) | (2.02 to 4.36) | (-0.09 to 0.01) | (-0.59 to -0.43) |
| Central Latin America | 116761 | 72.44 | 150580 | 60.05 | 0.29 | 0.36% | 4156 | 3.29 | 6582 | 2.64 | 0.58 | 0.33% |
|  | (83477 to 167528) | (51.59 to 103.67) | (106506 to 216032) | (42.47 to 86.17) | (0.24-0.34) | (0 to 0.71) | (2720 to 5994) | (2.21 to 4.69) | (4436 to 9388) | (1.78 to 3.76) | (0.51 to 0.67) | (0.01 to 0.65) |
| Central Sub-Saharan Africa | 10663 | 18.85 | 23719 | 18.16 | 1.22 | -0.11% | 356 | 0.9 | 818 | 0.85 | 1.3 | -0.20% |
|  | (7792 to 15451) | (13.99 to 26.03) | (17560 to 34145) | (13.63 to 24.81) | (1.11-1.33) | (-0.22 to 0) | (233 to 520) | (0.59 to 1.29) | (538 to 1179) | (0.56 to 1.24) | (1.12 to 1.5) | (-0.27 to -0.12) |
| East Asia | 693305 | 57.94 | 1159376 | 68.82 | 0.67 | 0.13% | 28293 | 2.65 | 55778 | 2.96 | 0.97 | -0.10% |
|  | (471617 to 1037809) | (39.69 to 86.87) | (782424 to 1732608) | (46.67 to 104.63) | (0.51-0.86) | (-0.16 to 0.43) | (18487 to 41291) | (1.75 to 3.82) | (36863 to 78918) | (1.94 to 4.22) | (0.86 to 1.08) | (-0.41 to 0.2) |
| Eastern Europe | 178754 | 78.78 | 133318 | 63.99 | -0.25 | -0.75% | 8498 | 3.39 | 7237 | 2.74 | -0.15 | -0.81% |
|  | (127301 to 248677) | (56.36 to 109.34) | (95134 to 185965) | (45.47 to 89.15) | (-0.29--0.22) | (-0.9 to -0.61) | (5713 to 12120) | (2.27 to 4.87) | (4913 to 10255) | (1.84 to 3.95) | (-0.19 to -0.1) | (-0.95 to -0.66) |
| Eastern Sub-Saharan Africa | 79487 | 46.81 | 171114 | 45.48 | 1.15 | -0.11% | 2693 | 2.23 | 5831 | 2.1 | 1.17 | -0.25% |
|  | (58529 to 108314) | (34.14 to 64.87) | (125700 to 231729) | (33.35 to 62.06) | (1.08-1.25) | (-0.19 to -0.02) | (1780 to 3853) | (1.5 to 3.15) | (3819 to 8456) | (1.4 to 2.99) | (1.08 to 1.26) | (-0.33 to -0.17) |
| High-income Asia Pacific | 138822 | 77.96 | 138336 | 69.14 | 0 | -0.54% | 6597 | 3.44 | 8558 | 3.03 | 0.3 | -0.55% |
|  | (96233 to 200093) | (54.02 to 112.01) | (94837 to 202877) | (47.12 to 100.74) | (-0.07-0.08) | (-0.64 to -0.45) | (4378 to 9522) | (2.29 to 4.95) | (5735 to 12126) | (2 to 4.39) | (0.23 to 0.37) | (-0.64 to -0.45) |
| High-income North America | 344930 | 118.19 | 464069 | 110.75 | 0.35 | -0.54% | 15779 | 5.02 | 22997 | 4.65 | 0.46 | -0.59% |
|  | (238883 to 505558) | (81.74 to 173.97) | (319927 to 700114) | (76.44 to 162.91) | (0.23-0.46) | (-0.8 to -0.29) | (10537 to 22343) | (3.34 to 7.17) | (15414 to 32459) | (3.11 to 6.62) | (0.39 to 0.52) | (-0.85 to -0.33) |
| High-middle SDI | 717091 | 61.54 | 917791 | 59.21 | 0.28 | -0.27% | 31363 | 2.76 | 45885 | 2.58 | 0.46 | -0.39% |
|  | (514923 to 1009719) | (44.13 to 86.57) | (646520 to 1330637) | (42.14 to 85.63) | (0.18-0.38) | (-0.36 to -0.19) | (20974 to 45226) | (1.85 to 3.97) | (30714 to 65465) | (1.7 to 3.69) | (0.41 to 0.52) | (-0.48 to -0.31) |
| High SDI | 683048 | 80.77 | 862165 | 76.4 | 0.26 | -0.40% | 31831 | 3.45 | 45066 | 3.23 | 0.42 | -0.43% |
|  | (475225 to 991868) | (56.37 to 116.23) | (594883 to 1280563) | (52.8 to 112.09) | (0.17-0.36) | (-0.55 to -0.24) | (21232 to 45320) | (2.29 to 4.94) | (30251 to 63922) | (2.16 to 4.63) | (0.37 to 0.47) | (-0.57 to -0.28) |
| Low-middle SDI | 400716 | 37.82 | 660441 | 38.56 | 0.65 | -0.32% | 15139 | 1.8 | 28406 | 1.81 | 0.88 | -0.18% |
|  | (298120 to 536906) | (28.16 to 50.5) | (488654 to 893946) | (28.47 to 52.5) | (0.55-0.76) | (-0.69 to 0.06) | (10010 to 21743) | (1.2 to 2.56) | (19196 to 40453) | (1.22 to 2.56) | (0.8 to 0.96) | (-0.31 to -0.06) |
| Low SDI | 173714 | 36.43 | 380748 | 37.26 | 1.19 | 0.10% | 6681 | 1.89 | 14600 | 1.86 | 1.19 | -0.04% |
|  | (129082 to 232377) | (27.04 to 49) | (282420 to 515180) | (27.58 to 50.16) | (1.11-1.28) | (-0.2 to 0.39) | (4425 to 9559) | (1.28 to 2.67) | (9793 to 20890) | (1.26 to 2.65) | (1.12 to 1.26) | (-0.13 to 0.04) |
| Middle SDI | 785382 | 47.42 | 1151125 | 47.16 | 0.47 | -0.18% | 32472 | 2.35 | 56805 | 2.26 | 0.75 | -0.36% |
|  | (570143 to 1101032) | (34.12 to 66.85) | (819070 to 1626403) | (33.54 to 66.82) | (0.37-0.57) | (-0.34 to -0.02) | (21379 to 46819) | (1.56 to 3.36) | (37927 to 81213) | (1.5 to 3.22) | (0.68 to 0.82) | (-0.55 to -0.16) |
| North Africa and Middle East | 123461 | 36.15 | 182371 | 29.3 | 0.48 | -0.22% | 3840 | 1.42 | 7494 | 1.31 | 0.95 | -0.15% |
|  | (91807 to 165002) | (26.68 to 48.93) | (132011 to 255381) | (21.27 to 40.79) | (0.16-0.72) | (-0.53 to 0.08) | (2528 to 5589) | (0.95 to 2.07) | (5004 to 10732) | (0.88 to 1.86) | (0.79 to 1.11) | (-0.26 to -0.05) |
| Oceania | 1242 | 20.41 | 2795 | 22.18 | 1.25 | -0.21% | 46 | 0.98 | 112 | 1.09 | 1.45 | 0.20% |
|  | (919 to 1647) | (15.06 to 26.87) | (2058 to 3802) | (16.38 to 29.98) | (1.12-1.4) | (-1.11 to 0.7) | (30 to 66) | (0.66 to 1.39) | (73 to 161) | (0.73 to 1.55) | (1.18 to 1.79) | (-0.15 to 0.55) |
| South Asia | 332043 | 32.69 | 575267 | 32.69 | 0.73 | -0.37% | 12387 | 1.52 | 24301 | 1.51 | 0.96 | -0.21% |
|  | (245059 to 442921) | (23.98 to 43.43) | (425099 to 777301) | (24.17 to 44.44) | (0.6-0.88) | (-0.75 to 0.01) | (8171 to 17854) | (1.02 to 2.17) | (16467 to 34751) | (1.02 to 2.14) | (0.88 to 1.06) | (-0.35 to -0.08) |
| Southeast Asia | 190903 | 42.37 | 247440 | 36.61 | 0.3 | -0.54% | 7144 | 1.96 | 11164 | 1.66 | 0.56 | -0.66% |
|  | (139809 to 260422) | (30.87 to 57.96) | (179861 to 340179) | (26.39 to 50.53) | (0.23-0.37) | (-0.91 to -0.17) | (4778 to 10288) | (1.32 to 2.79) | (7484 to 15861) | (1.12 to 2.36) | (0.49 to 0.65) | (-0.79 to -0.54) |
| Southern Latin America | 24121 | 48.5 | 31751 | 47.93 | 0.32 | -0.20% | 1057 | 2.21 | 1535 | 2.1 | 0.45 | -0.33% |
|  | (16567 to 35927) | (33.32 to 72.41) | (21886 to 46773) | (32.97 to 70.8) | (0.26-0.38) | (-0.28 to -0.12) | (691 to 1546) | (1.44 to 3.23) | (1006 to 2229) | (1.37 to 3.06) | (0.3 to 0.63) | (-0.4 to -0.25) |
| Southern Sub-Saharan Africa | 30118 | 60.66 | 40922 | 52.75 | 0.36 | -0.36% | 1123 | 2.85 | 1648 | 2.36 | 0.47 | -0.47% |
|  | (21722 to 41220) | (43.55 to 83.08) | (29819 to 55369) | (38.16 to 71.31) | (0.28-0.44) | (-0.52 to -0.19) | (746 to 1614) | (1.92 to 4.03) | (1102 to 2357) | (1.59 to 3.33) | (0.38 to 0.55) | (-0.66 to -0.29) |
| Tropical Latin America | 148350 | 98.36 | 195840 | 85.45 | 0.32 | -0.40% | 5569 | 4.45 | 9036 | 3.75 | 0.62 | -0.53% |
|  | (100191 to 219899) | (66.99 to 145.33) | (131930 to 295185) | (57.56 to 129.29) | (0.25-0.4) | (-0.46 to -0.35) | (3613 to 8187) | (2.93 to 6.38) | (5928 to 13004) | (2.46 to 5.4) | (0.53 to 0.72) | (-0.59 to -0.46) |
| Western Europe | 187504 | 47.92 | 204419 | 44.59 | 0.09 | -0.44% | 9243 | 2.03 | 11253 | 1.89 | 0.22 | -0.46% |
|  | (131716 to 269835) | (34 to 69.14) | (139437 to 303636) | (30.82 to 66.2) | (0.02-0.17) | (-0.52 to -0.37) | (6098 to 13252) | (1.33 to 2.92) | (7459 to 16085) | (1.24 to 2.76) | (0.16 to 0.28) | (-0.53 to -0.38) |
| Western Sub-Saharan Africa | 90123 | 54.04 | 214758 | 55.59 | 1.38 | 0.14% | 3199 | 2.54 | 7489 | 2.57 | 1.34 | 0.08% |
|  | (64446 to 125550) | (38.08 to 75.68) | (152982 to 297153) | (38.96 to 78.38) | (1.3-1.46) | (0.08 to 0.21) | (2104 to 4584) | (1.7 to 3.59) | (4906 to 10756) | (1.74 to 3.65) | (1.28 to 1.42) | (0.01 to 0.14) |
