# Supplementary material for: Global, regional, and national burden of fracture of sternum and/or fracture of one or more ribs: a systematic analysis of incidence, YLDs with projections to 2030
Source: Front Public Health. 2025 Apr 3;13:1565478. doi: 10.3389/fpubh.2025.1565478 (PMC12003264; doi:10.3389/fpubh.2025.1565478)
Supplement: Supplementary file 2 [file Table_2.docx]

| Table 2. The incidence and YLDs of sternum/ribs fractures in 1990 and 2019 and estimated annual percentage change from 1990 to 2019 across 204 countries. | | | | | | | | | | | | |
| --- | --- | --- | --- | --- | --- | --- | --- | --- | --- | --- | --- | --- |
|  | incidence | | | | | | YLDs | | | | | |
| location | Num_1990 (95% UI) | ASR_1990 (95% UI) | Num_2019 (95% UI) | ASR_2019 (95% UI) | Num_change | EAPC (95% CI) | Num_1990 (95% UI) | ASR_1990 (95% UI) | Num_2019 (95% UI) | ASR_2019 (95% UI) | Num_change | EAPC (95% CI) |
| Afghanistan | 2713 | 24.1 | 9150 | 24.29 | 2.37 | -1.06% | 106 | 1.2 | 324 | 1.23 | 2.05 | -0.37% |
|  | (1943 to 3827) | (17.27 to 34.21) | (6623 to 12657) | (17.54 to 33.26) | (2.16 to 2.61) | (-2.28 to 0.18) | (69 to 157) | (0.79 to 1.76) | (209 to 471) | (0.79 to 1.78) | (1.52 to 2.73) | (-0.86 to 0.13) |
| Albania | 2980 | 87.31 | 1873 | 71.39 | -0.37 | -1.09% | 115 | 3.99 | 102 | 3.12 | -0.11 | -1.26% |
|  | (1902 to 4933) | (55.89 to 145.01) | (1254 to 2943) | (47.55 to 111.08) | (-0.43 to -0.28) | (-1.25 to -0.93) | (74 to 172) | (2.61 to 5.91) | (66 to 149) | (2.04 to 4.54) | (-0.24 to 0.03) | (-1.43 to -1.09) |
| Algeria | 6727 | 26.2 | 10186 | 24.11 | 0.51 | -0.57% | 236 | 1.24 | 447 | 1.11 | 0.89 | -0.53% |
|  | (4841 to 9220) | (18.89 to 35.9) | (7352 to 14123) | (17.4 to 33.42) | (0.42 to 0.61) | (-1.02 to -0.12) | (155 to 342) | (0.82 to 1.79) | (294 to 654) | (0.74 to 1.61) | (0.61 to 1.27) | (-0.67 to -0.38) |
| American Samoa | 27 | 59.22 | 31 | 57.7 | 0.16 | 0.03% | 1 | 2.7 | 1 | 2.65 | 0.44 | -0.01% |
|  | (19 to 38) | (41.94 to 83.26) | (22 to 46) | (40.57 to 84.65) | (0.08 to 0.26) | (-0.72 to 0.8) | (1 to 1) | (1.77 to 3.94) | (1 to 2) | (1.77 to 3.84) | (0.24 to 0.67) | (-0.3 to 0.28) |
| Andorra | 22 | 41.76 | 36 | 42.65 | 0.63 | 0.03% | 1 | 1.79 | 2 | 1.83 | 1 | 0.01% |
|  | (16 to 32) | (29.18 to 60.91) | (25 to 54) | (29.01 to 63.61) | (0.51 to 0.76) | (-0.02 to 0.07) | (1 to 1) | (1.16 to 2.64) | (1 to 3) | (1.19 to 2.72) | (0.66 to 1.46) | (-0.04 to 0.07) |
| Angola | 2103 | 20.13 | 5411 | 18.16 | 1.57 | -0.40% | 71 | 0.97 | 179 | 0.85 | 1.53 | -0.53% |
|  | (1538 to 3021) | (15 to 28) | (3989 to 7743) | (13.48 to 24.66) | (1.43 to 1.73) | (-0.46 to -0.34) | (46 to 104) | (0.64 to 1.44) | (117 to 260) | (0.55 to 1.23) | (1.23 to 1.87) | (-0.58 to -0.48) |
| Antigua and Barbuda | 19 | 31.02 | 29 | 33.47 | 0.52 | -0.01% | 1 | 1.4 | 1 | 1.49 | 0.84 | 0.08% |
|  | (13 to 31) | (21.08 to 49.65) | (20 to 47) | (22.77 to 53.19) | (0.41 to 0.63) | (-0.41 to 0.38) | (1 to 1) | (0.91 to 2.03) | (1 to 2) | (0.95 to 2.18) | (0.53 to 1.21) | (-0.11 to 0.26) |
| Argentina | 15864 | 48.06 | 21234 | 47.33 | 0.34 | -0.22% | 708 | 2.19 | 1009 | 2.09 | 0.43 | -0.32% |
|  | (10922 to 23287) | (33.1 to 70.62) | (14595 to 31239) | (32.56 to 69.55) | (0.28 to 0.39) | (-0.3 to -0.13) | (455 to 1032) | (1.4 to 3.19) | (650 to 1489) | (1.34 to 3.06) | (0.21 to 0.68) | (-0.4 to -0.24) |
| Armenia | 1777 | 50.82 | 1050 | 36.41 | -0.41 | -1.30% | 91 | 2.84 | 62 | 1.79 | -0.32 | -1.73% |
|  | (1258 to 2549) | (36.06 to 72.85) | (735 to 1514) | (25.6 to 52.13) | (-0.44 to -0.37) | (-1.51 to -1.08) | (60 to 132) | (1.86 to 4.07) | (41 to 89) | (1.19 to 2.59) | (-0.45 to -0.17) | (-1.92 to -1.55) |
| Australia | 14073 | 84.27 | 19946 | 83.2 | 0.42 | -0.08% | 649 | 3.61 | 1055 | 3.54 | 0.63 | -0.12% |
|  | (9468 to 21847) | (56.59 to 130.03) | (13498 to 29985) | (56.02 to 126.05) | (0.33 to 0.53) | (-0.15 to -0.01) | (433 to 934) | (2.4 to 5.24) | (710 to 1522) | (2.35 to 5.18) | (0.44 to 0.86) | (-0.19 to -0.05) |
| Austria | 4024 | 50.04 | 3990 | 42.93 | -0.01 | -0.68% | 195 | 2.1 | 221 | 1.82 | 0.13 | -0.64% |
|  | (2770 to 5896) | (34.63 to 72.97) | (2705 to 5897) | (29.54 to 63.67) | (-0.08 to 0.06) | (-0.72 to -0.63) | (129 to 280) | (1.37 to 3.01) | (148 to 313) | (1.2 to 2.61) | (-0.04 to 0.33) | (-0.69 to -0.6) |
| Azerbaijan | 2933 | 37.85 | 3578 | 34.58 | 0.22 | -0.32% | 114 | 1.72 | 167 | 1.55 | 0.47 | -0.38% |
|  | (2084 to 4098) | (27.01 to 52.87) | (2525 to 5138) | (24.52 to 49.26) | (0.12 to 0.31) | (-0.54 to -0.1) | (74 to 168) | (1.12 to 2.53) | (106 to 243) | (1 to 2.26) | (0.22 to 0.78) | (-0.56 to -0.19) |
| Bahrain | 126 | 23.13 | 357 | 24.9 | 1.84 | 0.35% | 5 | 1.06 | 18 | 1.12 | 2.94 | 0.18% |
|  | (90 to 173) | (16.81 to 31.67) | (256 to 499) | (17.84 to 34.71) | (1.63 to 2.07) | (0.21 to 0.48) | (3 to 7) | (0.68 to 1.56) | (11 to 27) | (0.73 to 1.65) | (2.04 to 4.08) | (0.07 to 0.29) |
| Bangladesh | 23039 | 21.39 | 35576 | 21.96 | 0.54 | -1.42% | 812 | 1.03 | 1717 | 1.14 | 1.12 | -0.99% |
|  | (17412 to 30124) | (16.18 to 28.25) | (26245 to 47459) | (16.27 to 29.2) | (0.35 to 0.72) | (-3.21 to 0.4) | (531 to 1185) | (0.67 to 1.52) | (1160 to 2485) | (0.77 to 1.64) | (0.77 to 1.53) | (-1.85 to -0.13) |
| Barbados | 60 | 23.23 | 70 | 25.24 | 0.18 | 0.25% | 3 | 1.04 | 4 | 1.11 | 0.46 | 0.19% |
|  | (44 to 83) | (16.93 to 32.73) | (51 to 99) | (18.43 to 35.6) | (0.11 to 0.26) | (0.19 to 0.31) | (2 to 4) | (0.68 to 1.51) | (3 to 6) | (0.71 to 1.64) | (0.2 to 0.79) | (0.14 to 0.25) |
| Belarus | 6928 | 66.41 | 5943 | 62.86 | -0.14 | 0.02% | 331 | 2.88 | 321 | 2.66 | -0.03 | -0.10% |
|  | (4991 to 9604) | (47.93 to 91.48) | (4236 to 8403) | (45.22 to 89.24) | (-0.2 to -0.08) | (-0.18 to 0.21) | (219 to 480) | (1.9 to 4.18) | (213 to 463) | (1.77 to 3.87) | (-0.16 to 0.11) | (-0.28 to 0.09) |
| Belgium | 4602 | 45.72 | 6009 | 48.49 | 0.31 | 0.75% | 232 | 1.95 | 321 | 2.05 | 0.38 | 0.69% |
|  | (3174 to 6666) | (31.86 to 66.86) | (4052 to 8981) | (33.21 to 71.79) | (0.19 to 0.43) | (0.33 to 1.17) | (153 to 337) | (1.27 to 2.82) | (213 to 462) | (1.34 to 2.99) | (0.21 to 0.6) | (0.29 to 1.1) |
| Belize | 50 | 25.56 | 133 | 31.59 | 1.68 | 0.53% | 2 | 1.19 | 5 | 1.46 | 2.15 | 0.61% |
|  | (36 to 71) | (18.42 to 37.02) | (95 to 192) | (22.68 to 45.39) | (1.48 to 1.89) | (0.12 to 0.94) | (1 to 3) | (0.78 to 1.77) | (3 to 8) | (0.96 to 2.16) | (1.71 to 2.67) | (0.39 to 0.83) |
| Benin | 2410 | 59.24 | 6063 | 58.1 | 1.52 | -0.12% | 83 | 2.8 | 208 | 2.69 | 1.51 | -0.18% |
|  | (1703 to 3426) | (40.83 to 85.84) | (4288 to 8454) | (40.64 to 82.7) | (1.38 to 1.65) | (-0.15 to -0.08) | (54 to 119) | (1.86 to 3.99) | (134 to 309) | (1.78 to 3.92) | (1.21 to 1.84) | (-0.22 to -0.15) |
| Bermuda | 38 | 63.07 | 45 | 69.48 | 0.19 | 0.30% | 2 | 2.76 | 3 | 2.98 | 0.49 | 0.27% |
|  | (27 to 54) | (44.64 to 89.07) | (31 to 66) | (49.04 to 98.83) | (0.04 to 0.36) | (0.2 to 0.41) | (1 to 3) | (1.81 to 4.01) | (2 to 4) | (1.95 to 4.39) | (0.28 to 0.76) | (0.18 to 0.36) |
| Bhutan | 154 | 26.21 | 225 | 29.54 | 0.46 | 0.10% | 5 | 1.25 | 9 | 1.37 | 0.77 | 0.18% |
|  | (113 to 211) | (19.16 to 35.69) | (163 to 300) | (21.58 to 39.49) | (0.33 to 0.63) | (-0.49 to 0.69) | (3 to 8) | (0.8 to 1.81) | (6 to 14) | (0.91 to 1.97) | (0.47 to 1.11) | (-0.02 to 0.37) |
| Bosnia and Herzegovina | 3122 | 67.61 | 2065 | 67.37 | -0.34 | 0.10% | 138 | 2.99 | 120 | 2.89 | -0.13 | -0.04% |
|  | (2114 to 4671) | (46.12 to 100.72) | (1407 to 3092) | (45.91 to 100.37) | (-0.38 to -0.29) | (0.01 to 0.2) | (89 to 202) | (1.94 to 4.38) | (79 to 174) | (1.89 to 4.26) | (-0.25 to 0.03) | (-0.13 to 0.06) |
| Botswana | 618 | 49.92 | 1261 | 54.93 | 1.04 | 0.21% | 21 | 2.3 | 48 | 2.43 | 1.29 | 0.07% |
|  | (446 to 851) | (36.02 to 68.96) | (908 to 1734) | (39.32 to 76.13) | (0.87 to 1.27) | (0.11 to 0.31) | (14 to 31) | (1.51 to 3.34) | (31 to 71) | (1.56 to 3.51) | (0.95 to 1.68) | (-0.02 to 0.17) |
| Brazil | 147205 | 100.21 | 193913 | 87.3 | 0.32 | -0.39% | 5527 | 4.53 | 8956 | 3.82 | 0.62 | -0.53% |
|  | (99205 to 218589) | (68.19 to 148.1) | (130579 to 292444) | (58.7 to 132.34) | (0.24 to 0.4) | (-0.45 to -0.34) | (3583 to 8130) | (2.98 to 6.5) | (5877 to 12886) | (2.51 to 5.5) | (0.53 to 0.71) | (-0.59 to -0.46) |
| Bulgaria | 6813 | 81.63 | 4660 | 73.46 | -0.32 | -0.32% | 361 | 3.6 | 289 | 3.17 | -0.2 | -0.41% |
|  | (4750 to 9807) | (56.88 to 116.77) | (3112 to 7116) | (49.33 to 110.71) | (-0.38 to -0.25) | (-0.36 to -0.29) | (237 to 521) | (2.35 to 5.21) | (192 to 421) | (2.07 to 4.73) | (-0.3 to -0.07) | (-0.44 to -0.38) |
| Burkina Faso | 4060 | 50.52 | 11252 | 59.35 | 1.77 | 0.46% | 143 | 2.39 | 371 | 2.63 | 1.6 | 0.37% |
|  | (2896 to 5676) | (35.71 to 70.79) | (8104 to 15326) | (41.95 to 82.1) | (1.57 to 2.13) | (0.31 to 0.61) | (93 to 205) | (1.57 to 3.41) | (239 to 540) | (1.75 to 3.82) | (1.29 to 1.97) | (0.22 to 0.51) |
| Burundi | 1246 | 23.56 | 2571 | 22.27 | 1.06 | -0.50% | 43 | 1.13 | 88 | 1.07 | 1.07 | -0.36% |
|  | (922 to 1679) | (17.51 to 32.08) | (1918 to 3515) | (16.63 to 30.21) | (0.98 to 1.15) | (-1.25 to 0.26) | (28 to 62) | (0.75 to 1.64) | (58 to 129) | (0.71 to 1.54) | (0.79 to 1.37) | (-0.64 to -0.08) |
| Cambodia | 1974 | 20.27 | 3695 | 22.72 | 0.87 | 0.20% | 69 | 0.97 | 150 | 1.03 | 1.19 | 0.09% |
|  | (1441 to 2647) | (14.89 to 27.14) | (2672 to 5086) | (16.43 to 31.27) | (0.7 to 1.09) | (-0.01 to 0.4) | (44 to 103) | (0.63 to 1.41) | (98 to 221) | (0.67 to 1.49) | (0.86 to 1.55) | (-0.02 to 0.19) |
| Cameroon | 5226 | 60.61 | 15391 | 63.07 | 1.94 | 0.18% | 186 | 2.88 | 533 | 2.87 | 1.87 | 0.04% |
|  | (3639 to 7481) | (41.26 to 88.06) | (10764 to 21778) | (43.43 to 90.72) | (1.78 to 2.13) | (0.13 to 0.23) | (121 to 266) | (1.91 to 4.07) | (348 to 761) | (1.9 to 4.03) | (1.49 to 2.29) | (0.01 to 0.08) |
| Canada | 11444 | 40.21 | 16230 | 38.56 | 0.42 | -0.17% | 522 | 1.73 | 849 | 1.65 | 0.63 | -0.19% |
|  | (8252 to 16117) | (28.73 to 56.67) | (11422 to 23208) | (27.34 to 54.26) | (0.29 to 0.57) | (-0.22 to -0.12) | (338 to 758) | (1.11 to 2.51) | (555 to 1225) | (1.07 to 2.43) | (0.36 to 0.93) | (-0.24 to -0.14) |
| Central African Republic | 500 | 18.18 | 1011 | 19.21 | 1.02 | 0.68% | 17 | 0.87 | 37 | 0.94 | 1.12 | 0.41% |
|  | (368 to 716) | (13.6 to 24.99) | (752 to 1398) | (14.42 to 25.92) | (0.9 to 1.2) | (0.29 to 1.07) | (11 to 25) | (0.58 to 1.26) | (24 to 53) | (0.62 to 1.37) | (0.83 to 1.48) | (0.26 to 0.55) |
| Chad | 2576 | 49.54 | 7414 | 56.15 | 1.88 | 0.50% | 91 | 2.35 | 246 | 2.68 | 1.69 | 0.53% |
|  | (1829 to 3622) | (34.96 to 69.44) | (5266 to 10308) | (39.06 to 78.81) | (1.77 to 1.98) | (0.45 to 0.54) | (59 to 131) | (1.55 to 3.34) | (158 to 358) | (1.75 to 3.85) | (1.39 to 2.03) | (0.49 to 0.57) |
| Chile | 6283 | 46.34 | 8665 | 48.01 | 0.38 | 0.01% | 253 | 2.08 | 430 | 2.08 | 0.7 | -0.15% |
|  | (4358 to 9244) | (32.22 to 67.69) | (5990 to 12699) | (32.99 to 70.89) | (0.3 to 0.47) | (-0.09 to 0.12) | (166 to 370) | (1.37 to 3.03) | (278 to 616) | (1.34 to 2.98) | (0.43 to 1.02) | (-0.22 to -0.08) |
| China | 686130 | 59.41 | 1151480 | 70.82 | 0.68 | 0.14% | 27998 | 2.72 | 55366 | 3.05 | 0.98 | -0.10% |
|  | (466014 to 1027496) | (40.62 to 89.14) | (776788 to 1722025) | (47.98 to 107.85) | (0.51 to 0.86) | (-0.16 to 0.44) | (18292 to 40880) | (1.8 to 3.92) | (36613 to 78375) | (2 to 4.34) | (0.87 to 1.09) | (-0.41 to 0.21) |
| Colombia | 13945 | 41.46 | 14219 | 30.18 | 0.02 | -1.50% | 529 | 1.96 | 676 | 1.34 | 0.28 | -1.70% |
|  | (9780 to 19769) | (29.4 to 58.2) | (10253 to 19298) | (21.71 to 41.13) | (-0.17 to 0.12) | (-1.73 to -1.28) | (350 to 761) | (1.31 to 2.78) | (443 to 986) | (0.88 to 1.96) | (0.07 to 0.56) | (-1.9 to -1.5) |
| Comoros | 297 | 72.02 | 483 | 70.92 | 0.63 | -0.18% | 11 | 3.45 | 20 | 3.29 | 0.84 | -0.32% |
|  | (210 to 414) | (49.79 to 101.97) | (341 to 681) | (49.32 to 100) | (0.54 to 0.73) | (-0.29 to -0.08) | (7 to 15) | (2.25 to 4.98) | (13 to 28) | (2.2 to 4.69) | (0.62 to 1.11) | (-0.44 to -0.2) |
| Congo | 450 | 18.56 | 896 | 17.22 | 0.99 | -0.26% | 15 | 0.88 | 34 | 0.81 | 1.22 | -0.32% |
|  | (333 to 646) | (13.78 to 25.56) | (667 to 1259) | (12.82 to 23.6) | (0.91 to 1.08) | (-0.39 to -0.14) | (10 to 23) | (0.57 to 1.27) | (22 to 50) | (0.54 to 1.2) | (0.96 to 1.53) | (-0.42 to -0.22) |
| Cook Islands | 11 | 61.37 | 11 | 61.28 | 0.03 | -0.27% | 0 | 2.68 | 1 | 2.75 | 0.38 | -0.10% |
|  | (8 to 15) | (43.25 to 86.4) | (8 to 17) | (41.66 to 93.53) | (-0.08 to 0.15) | (-1.14 to 0.61) | (0 to 1) | (1.77 to 3.83) | (0 to 1) | (1.82 to 3.98) | (0.18 to 0.61) | (-0.45 to 0.26) |
| Costa Rica | 931 | 30.2 | 1427 | 30.89 | 0.53 | 0.06% | 33 | 1.32 | 66 | 1.32 | 0.96 | 0.02% |
|  | (680 to 1281) | (22.06 to 40.9) | (1032 to 1935) | (22.25 to 42.11) | (0.44 to 0.64) | (-0.05 to 0.17) | (22 to 49) | (0.86 to 1.94) | (43 to 96) | (0.87 to 1.93) | (0.68 to 1.29) | (-0.07 to 0.1) |
| Croatia | 4045 | 82.38 | 3109 | 69.09 | -0.23 | -0.47% | 195 | 3.5 | 170 | 2.9 | -0.13 | -0.51% |
|  | (2808 to 5801) | (57.15 to 119.85) | (2132 to 4572) | (47.95 to 100.72) | (-0.34 to -0.09) | (-0.57 to -0.36) | (127 to 281) | (2.26 to 5.06) | (113 to 243) | (1.93 to 4.19) | (-0.26 to 0.02) | (-0.63 to -0.4) |
| Cuba | 3383 | 30.63 | 4313 | 36.75 | 0.28 | 0.70% | 146 | 1.35 | 226 | 1.55 | 0.55 | 0.55% |
|  | (2515 to 4585) | (22.66 to 41.41) | (3068 to 6076) | (26.4 to 50.78) | (0.15 to 0.42) | (0.63 to 0.77) | (93 to 218) | (0.86 to 2.02) | (148 to 325) | (1.02 to 2.26) | (0.28 to 0.88) | (0.48 to 0.62) |
| Cyprus | 322 | 41.79 | 534 | 41.39 | 0.66 | -0.10% | 14 | 1.8 | 27 | 1.75 | 0.92 | -0.14% |
|  | (225 to 468) | (29.22 to 60.66) | (370 to 789) | (28.69 to 61.6) | (0.52 to 0.82) | (-0.16 to -0.04) | (9 to 21) | (1.16 to 2.64) | (18 to 40) | (1.14 to 2.53) | (0.64 to 1.27) | (-0.2 to -0.09) |
| Democratic Republic of the Congo | 7324 | 18.52 | 15822 | 18.14 | 1.16 | -0.06% | 243 | 0.88 | 547 | 0.85 | 1.25 | -0.13% |
|  | (5327 to 10688) | (13.73 to 25.44) | (11674 to 22704) | (13.63 to 24.73) | (1.04 to 1.28) | (-0.22 to 0.09) | (157 to 357) | (0.58 to 1.27) | (355 to 794) | (0.55 to 1.24) | (1.01 to 1.52) | (-0.21 to -0.05) |
| Denmark | 2415 | 43.81 | 2264 | 39.12 | -0.06 | -0.55% | 117 | 1.84 | 125 | 1.67 | 0.07 | -0.47% |
|  | (1662 to 3506) | (30.49 to 63.82) | (1545 to 3351) | (26.92 to 58.47) | (-0.11 to -0.02) | (-0.64 to -0.46) | (79 to 171) | (1.21 to 2.66) | (81 to 184) | (1.05 to 2.47) | (-0.1 to 0.27) | (-0.56 to -0.38) |
| Djibouti | 275 | 65.31 | 723 | 65.8 | 1.63 | -0.14% | 9 | 3.09 | 28 | 3.05 | 2.08 | -0.13% |
|  | (199 to 385) | (46.21 to 90.76) | (519 to 1002) | (46.84 to 90.96) | (1.5 to 1.76) | (-0.37 to 0.09) | (6 to 13) | (2.06 to 4.43) | (18 to 40) | (2 to 4.35) | (1.61 to 2.61) | (-0.2 to -0.05) |
| Dominica | 17 | 23.06 | 17 | 25.24 | -0.03 | 1.02% | 1 | 1.04 | 1 | 1.22 | 0.3 | 0.77% |
|  | (13 to 24) | (16.98 to 31.99) | (12 to 24) | (18.44 to 35.28) | (-0.09 to 0.03) | (0.33 to 1.71) | (0 to 1) | (0.68 to 1.52) | (1 to 1) | (0.82 to 1.76) | (0.11 to 0.58) | (0.53 to 1.02) |
| Dominican Republic | 1779 | 23.59 | 3336 | 30.18 | 0.88 | 0.88% | 65 | 1.12 | 145 | 1.38 | 1.23 | 0.84% |
|  | (1320 to 2462) | (17.53 to 32.88) | (2422 to 4726) | (21.95 to 42.61) | (0.73 to 1.01) | (0.6 to 1.16) | (42 to 95) | (0.74 to 1.62) | (95 to 210) | (0.91 to 1.98) | (0.85 to 1.69) | (0.71 to 0.98) |
| Ecuador | 2867 | 27.86 | 5112 | 28.86 | 0.78 | 0.07% | 110 | 1.37 | 222 | 1.32 | 1.02 | -0.15% |
|  | (2123 to 3959) | (20.4 to 38.65) | (3723 to 6998) | (21.06 to 39.44) | (0.65 to 0.93) | (-0.1 to 0.23) | (73 to 159) | (0.91 to 1.98) | (144 to 325) | (0.85 to 1.92) | (0.7 to 1.39) | (-0.23 to -0.07) |
| Egypt | 10738 | 18.61 | 20130 | 19.93 | 0.87 | 0.26% | 398 | 0.89 | 808 | 0.92 | 1.03 | 0.15% |
|  | (7824 to 14653) | (13.52 to 25.46) | (14524 to 27891) | (14.45 to 27.53) | (0.76 to 1) | (0.02 to 0.5) | (257 to 591) | (0.57 to 1.29) | (523 to 1183) | (0.61 to 1.35) | (0.72 to 1.37) | (0.02 to 0.28) |
| El Salvador | 1717 | 31.32 | 2161 | 34.05 | 0.26 | 0.12% | 63 | 1.46 | 93 | 1.51 | 0.49 | 0.05% |
|  | (1245 to 2283) | (22.75 to 41.6) | (1506 to 3004) | (23.69 to 47.28) | (0.14 to 0.41) | (-0.35 to 0.59) | (41 to 91) | (0.97 to 2.14) | (61 to 138) | (0.98 to 2.25) | (0.29 to 0.72) | (-0.14 to 0.23) |
| Equatorial Guinea | 86 | 19.34 | 252 | 17.84 | 1.93 | -0.25% | 3 | 0.93 | 8 | 0.81 | 1.87 | -0.51% |
|  | (63 to 124) | (14.4 to 26.92) | (186 to 355) | (13.3 to 24.82) | (1.64 to 2.21) | (-0.39 to -0.12) | (2 to 4) | (0.61 to 1.36) | (5 to 12) | (0.53 to 1.15) | (1.57 to 2.22) | (-0.65 to -0.38) |
| Eritrea | 1652 | 64.21 | 3939 | 66.02 | 1.39 | 0.04% | 54 | 3.01 | 137 | 3.03 | 1.52 | -0.04% |
|  | (1199 to 2315) | (45.37 to 88.93) | (2847 to 5446) | (46.99 to 92.07) | (1.27 to 1.51) | (0 to 0.08) | (35 to 80) | (2.04 to 4.35) | (88 to 203) | (2 to 4.37) | (1.17 to 1.91) | (-0.08 to 0) |
| Estonia | 1255 | 79.79 | 707 | 56.46 | -0.44 | -1.39% | 60 | 3.4 | 41 | 2.41 | -0.32 | -1.42% |
|  | (902 to 1748) | (57.24 to 110.9) | (502 to 1007) | (40.26 to 80.3) | (-0.48 to -0.4) | (-1.49 to -1.28) | (39 to 87) | (2.22 to 4.98) | (26 to 59) | (1.56 to 3.54) | (-0.42 to -0.2) | (-1.53 to -1.31) |
| Ethiopia | 13406 | 27.75 | 23875 | 22.71 | 0.78 | -0.69% | 453 | 1.34 | 816 | 1.07 | 0.8 | -0.93% |
|  | (9926 to 18561) | (20.76 to 37.96) | (17396 to 33764) | (16.9 to 30.74) | (0.7 to 0.87) | (-0.91 to -0.48) | (303 to 660) | (0.9 to 1.93) | (539 to 1185) | (0.72 to 1.53) | (0.69 to 0.93) | (-1.07 to -0.79) |
| Fiji | 124 | 16.25 | 150 | 16.56 | 0.22 | 0.11% | 5 | 0.78 | 7 | 0.8 | 0.47 | 0% |
|  | (90 to 167) | (11.93 to 21.88) | (111 to 204) | (12.22 to 22.48) | (0.14 to 0.3) | (-0.33 to 0.55) | (3 to 7) | (0.54 to 1.15) | (5 to 10) | (0.53 to 1.14) | (0.25 to 0.73) | (-0.15 to 0.16) |
| Finland | 3025 | 59.58 | 3161 | 55.21 | 0.05 | -0.25% | 146 | 2.49 | 177 | 2.33 | 0.22 | -0.22% |
|  | (2110 to 4343) | (41.72 to 86.19) | (2136 to 4714) | (37.55 to 82.51) | (-0.03 to 0.11) | (-0.65 to 0.15) | (95 to 211) | (1.61 to 3.63) | (117 to 257) | (1.5 to 3.46) | (0.05 to 0.41) | (-0.62 to 0.18) |
| France | 29465 | 49.05 | 33137 | 45.75 | 0.12 | -0.33% | 1395 | 2.07 | 1777 | 1.94 | 0.27 | -0.30% |
|  | (20523 to 42516) | (34.29 to 70.89) | (22450 to 49122) | (31.43 to 67.47) | (0.05 to 0.2) | (-0.36 to -0.29) | (906 to 1992) | (1.33 to 2.96) | (1159 to 2561) | (1.24 to 2.85) | (0.08 to 0.51) | (-0.34 to -0.27) |
| Gabon | 199 | 20.14 | 328 | 19.21 | 0.65 | -0.19% | 7 | 0.94 | 13 | 0.88 | 0.79 | -0.24% |
|  | (147 to 281) | (14.99 to 27.32) | (244 to 454) | (14.32 to 26.26) | (0.57 to 0.73) | (-0.26 to -0.11) | (5 to 10) | (0.61 to 1.37) | (8 to 19) | (0.57 to 1.28) | (0.57 to 1.08) | (-0.29 to -0.19) |
| Georgia | 2659 | 48.59 | 1773 | 51.11 | -0.33 | 0.36% | 124 | 2.13 | 97 | 2.21 | -0.22 | 0.32% |
|  | (1900 to 3768) | (34.74 to 68.85) | (1232 to 2595) | (35.85 to 74.23) | (-0.37 to -0.3) | (0.13 to 0.58) | (79 to 178) | (1.36 to 3.09) | (63 to 142) | (1.43 to 3.26) | (-0.34 to -0.07) | (0.12 to 0.53) |
| Germany | 32704 | 40.67 | 35374 | 39.88 | 0.08 | -0.21% | 1667 | 1.72 | 2020 | 1.7 | 0.21 | -0.19% |
|  | (22741 to 47333) | (28.58 to 59.09) | (24053 to 52575) | (27.29 to 59) | (0.01 to 0.16) | (-0.32 to -0.09) | (1069 to 2403) | (1.1 to 2.5) | (1316 to 2953) | (1.09 to 2.52) | (0.03 to 0.45) | (-0.3 to -0.09) |
| Ghana | 7139 | 55.41 | 18073 | 64.16 | 1.53 | 0.54% | 250 | 2.61 | 671 | 2.93 | 1.69 | 0.45% |
|  | (5007 to 10181) | (38.14 to 78.54) | (12480 to 25745) | (43.94 to 92.29) | (1.38 to 1.69) | (0.46 to 0.62) | (162 to 362) | (1.74 to 3.78) | (432 to 980) | (1.92 to 4.16) | (1.37 to 2.08) | (0.38 to 0.52) |
| Greece | 4158 | 41.16 | 3616 | 37.42 | -0.13 | -0.44% | 213 | 1.78 | 228 | 1.63 | 0.07 | -0.41% |
|  | (2949 to 5958) | (28.87 to 59.39) | (2508 to 5284) | (26.02 to 55.56) | (-0.18 to -0.08) | (-0.5 to -0.38) | (142 to 310) | (1.18 to 2.63) | (149 to 328) | (1.03 to 2.39) | (-0.09 to 0.27) | (-0.46 to -0.36) |
| Greenland | 100 | 186.96 | 86 | 143.55 | -0.14 | -1.06% | 4 | 8.21 | 4 | 6.18 | 0.01 | -1.12% |
|  | (67 to 148) | (125.46 to 277.84) | (57 to 133) | (95.28 to 220.2) | (-0.22 to -0.06) | (-1.12 to -0.99) | (3 to 6) | (5.37 to 11.7) | (3 to 6) | (4.04 to 8.87) | (-0.09 to 0.14) | (-1.18 to -1.06) |
| Grenada | 23 | 26.42 | 32 | 31.67 | 0.42 | 0.57% | 1 | 1.21 | 2 | 1.42 | 0.72 | 0.54% |
|  | (17 to 33) | (18.96 to 39.12) | (23 to 48) | (22.27 to 47.24) | (0.3 to 0.53) | (0.2 to 0.94) | (1 to 1) | (0.79 to 1.77) | (1 to 2) | (0.92 to 2.06) | (0.45 to 1.07) | (0.42 to 0.67) |
| Guam | 77 | 56.89 | 97 | 57.18 | 0.27 | -0.09% | 3 | 2.55 | 5 | 2.55 | 0.6 | -0.08% |
|  | (54 to 109) | (39.97 to 80.11) | (67 to 144) | (39.64 to 84.22) | (0.16 to 0.39) | (-0.18 to -0.01) | (2 to 4) | (1.69 to 3.72) | (3 to 7) | (1.67 to 3.72) | (0.36 to 0.87) | (-0.16 to 0.01) |
| Guatemala | 3209 | 37.2 | 6920 | 38.28 | 1.16 | 0.14% | 108 | 1.75 | 261 | 1.71 | 1.43 | -0.04% |
|  | (2334 to 4411) | (26.88 to 50.47) | (4828 to 10152) | (26.65 to 55.75) | (0.79 to 1.51) | (0.02 to 0.27) | (69 to 158) | (1.14 to 2.51) | (171 to 390) | (1.12 to 2.52) | (1.07 to 1.88) | (-0.11 to 0.02) |
| Guinea | 2952 | 53.39 | 6083 | 57.76 | 1.06 | 0.35% | 110 | 2.56 | 220 | 2.76 | 1 | 0.36% |
|  | (2088 to 4165) | (37.27 to 75.58) | (4297 to 8485) | (40.1 to 82.19) | (0.97 to 1.17) | (0.23 to 0.48) | (71 to 159) | (1.66 to 3.66) | (143 to 319) | (1.82 to 3.93) | (0.75 to 1.29) | (0.3 to 0.43) |
| Guinea-Bissau | 540 | 63.02 | 995 | 60.8 | 0.84 | -0.12% | 19 | 3.02 | 35 | 2.87 | 0.85 | -0.19% |
|  | (380 to 768) | (43.29 to 90.47) | (692 to 1406) | (41.94 to 86.5) | (0.76 to 0.93) | (-0.16 to -0.09) | (12 to 27) | (1.98 to 4.35) | (22 to 50) | (1.89 to 4.1) | (0.6 to 1.1) | (-0.23 to -0.15) |
| Guyana | 236 | 29.52 | 256 | 33 | 0.09 | 0.26% | 8 | 1.36 | 11 | 1.5 | 0.29 | 0.21% |
|  | (177 to 318) | (22.08 to 39.69) | (191 to 347) | (24.54 to 44.65) | (-0.01 to 0.18) | (0.11 to 0.4) | (5 to 13) | (0.87 to 1.98) | (7 to 16) | (0.98 to 2.18) | (0.09 to 0.56) | (0.13 to 0.29) |
| Haiti | 1853 | 28.76 | 3507 | 27.81 | 0.89 | 0.65% | 69 | 1.41 | 255 | 2.47 | 2.68 | 3.23% |
|  | (1337 to 2748) | (20.59 to 42.69) | (2518 to 5181) | (20.01 to 41.11) | (0.8 to 0.99) | (-2.38 to 3.76) | (44 to 103) | (0.91 to 2.06) | (149 to 421) | (1.48 to 4.13) | (1.39 to 5.89) | (1.36 to 5.14) |
| Honduras | 1620 | 32.55 | 3381 | 33.54 | 1.09 | -0.79% | 54 | 1.53 | 141 | 1.68 | 1.62 | -0.05% |
|  | (1143 to 2246) | (22.84 to 45.9) | (2336 to 4698) | (23.23 to 46.74) | (0.89 to 1.36) | (-2.5 to 0.94) | (34 to 79) | (0.98 to 2.24) | (93 to 203) | (1.11 to 2.38) | (1.2 to 2.24) | (-0.92 to 0.83) |
| Hungary | 8845 | 82.46 | 6581 | 68.86 | -0.26 | -0.85% | 429 | 3.51 | 375 | 2.92 | -0.13 | -0.83% |
|  | (6102 to 12855) | (57.47 to 118.9) | (4490 to 9699) | (47.3 to 101.8) | (-0.29 to -0.22) | (-0.96 to -0.74) | (284 to 611) | (2.31 to 5.05) | (247 to 541) | (1.91 to 4.27) | (-0.24 to 0) | (-0.95 to -0.72) |
| Iceland | 104 | 40.67 | 137 | 39.75 | 0.31 | -0.26% | 5 | 1.75 | 7 | 1.71 | 0.53 | -0.22% |
|  | (73 to 150) | (28.39 to 58.88) | (94 to 203) | (27.24 to 59.02) | (0.23 to 0.4) | (-0.43 to -0.09) | (3 to 7) | (1.12 to 2.58) | (5 to 10) | (1.1 to 2.5) | (0.3 to 0.83) | (-0.31 to -0.13) |
| India | 281494 | 35.71 | 480135 | 35.13 | 0.71 | -0.13% | 10562 | 1.66 | 20314 | 1.59 | 0.92 | -0.19% |
|  | (207014 to 376368) | (26.11 to 47.64) | (353108 to 655345) | (25.96 to 48.14) | (0.57 to 0.85) | (-0.24 to -0.02) | (6963 to 15234) | (1.11 to 2.36) | (13729 to 29135) | (1.08 to 2.27) | (0.83 to 1.02) | (-0.28 to -0.11) |
| Indonesia | 119936 | 68.19 | 149835 | 58.68 | 0.25 | -0.68% | 4534 | 3.19 | 6629 | 2.67 | 0.46 | -0.78% |
|  | (84832 to 170706) | (48.28 to 96.75) | (105495 to 211614) | (41.19 to 83.03) | (0.18 to 0.31) | (-0.89 to -0.46) | (2999 to 6571) | (2.15 to 4.58) | (4408 to 9600) | (1.79 to 3.82) | (0.39 to 0.55) | (-0.88 to -0.68) |
| Iraq | 6787 | 39.32 | 15444 | 35 | 1.28 | -0.45% | 230 | 1.83 | 568 | 1.58 | 1.47 | -0.57% |
|  | (4646 to 10212) | (26.71 to 59.81) | (10591 to 22611) | (24.05 to 51.35) | (1.1 to 1.46) | (-0.51 to -0.39) | (148 to 345) | (1.21 to 2.7) | (361 to 836) | (1.01 to 2.27) | (1.08 to 1.92) | (-0.6 to -0.54) |
| Ireland | 1457 | 40.76 | 1875 | 39.47 | 0.29 | -0.25% | 64 | 1.75 | 97 | 1.69 | 0.51 | -0.23% |
|  | (1014 to 2103) | (28.23 to 58.96) | (1283 to 2787) | (27.18 to 59.11) | (0.18 to 0.4) | (-0.33 to -0.16) | (42 to 95) | (1.13 to 2.58) | (63 to 143) | (1.1 to 2.48) | (0.27 to 0.78) | (-0.32 to -0.15) |
| Israel | 1892 | 37.69 | 3386 | 36.7 | 0.79 | -0.17% | 78 | 1.63 | 155 | 1.58 | 0.98 | -0.17% |
|  | (1313 to 2682) | (26.25 to 53.42) | (2334 to 4946) | (25.27 to 53.64) | (0.66 to 0.93) | (-0.23 to -0.1) | (51 to 115) | (1.07 to 2.37) | (100 to 225) | (1.02 to 2.3) | (0.65 to 1.35) | (-0.23 to -0.11) |
| Italy | 26693 | 45.77 | 21473 | 34.3 | -0.2 | -1.90% | 1335 | 1.94 | 1267 | 1.46 | -0.05 | -1.89% |
|  | (18702 to 38362) | (32.2 to 65.1) | (14771 to 32103) | (24 to 49.55) | (-0.27 to -0.12) | (-2.29 to -1.51) | (887 to 1914) | (1.27 to 2.78) | (831 to 1802) | (0.95 to 2.11) | (-0.11 to 0.01) | (-2.29 to -1.49) |
| Jamaica | 729 | 29.87 | 811 | 28.82 | 0.11 | -0.34% | 28 | 1.36 | 38 | 1.3 | 0.35 | -0.37% |
|  | (512 to 1102) | (20.93 to 44.98) | (569 to 1218) | (20.27 to 42.57) | (0 to 0.21) | (-0.45 to -0.23) | (18 to 42) | (0.87 to 1.99) | (25 to 57) | (0.84 to 1.93) | (0.14 to 0.65) | (-0.48 to -0.27) |
| Japan | 112633 | 87.45 | 109742 | 80.27 | -0.03 | -0.39% | 5573 | 3.77 | 6973 | 3.49 | 0.25 | -0.36% |
|  | (77141 to 165026) | (59.71 to 126.91) | (74804 to 162822) | (54.53 to 118.67) | (-0.1 to 0.07) | (-0.5 to -0.29) | (3738 to 7938) | (2.53 to 5.38) | (4695 to 9844) | (2.3 to 5.09) | (0.19 to 0.31) | (-0.46 to -0.25) |
| Jordan | 896 | 22.46 | 2677 | 22.08 | 1.99 | -0.17% | 29 | 1.04 | 102 | 0.99 | 2.54 | -0.34% |
|  | (648 to 1221) | (16.2 to 30.46) | (1918 to 3729) | (15.91 to 30.53) | (1.77 to 2.21) | (-0.27 to -0.06) | (19 to 43) | (0.69 to 1.55) | (66 to 153) | (0.64 to 1.46) | (2.02 to 3.08) | (-0.44 to -0.24) |
| Kazakhstan | 8097 | 48.23 | 8318 | 44.82 | 0.03 | -0.08% | 324 | 2.12 | 364 | 1.95 | 0.12 | -0.15% |
|  | (5737 to 11387) | (34.17 to 68.61) | (5927 to 11886) | (31.96 to 63.86) | (-0.02 to 0.07) | (-0.17 to 0.02) | (213 to 473) | (1.4 to 3.1) | (238 to 531) | (1.27 to 2.83) | (-0.05 to 0.35) | (-0.25 to -0.05) |
| Kenya | 5343 | 23.4 | 11362 | 23.24 | 1.13 | 0.02% | 171 | 1.09 | 409 | 1.07 | 1.39 | -0.08% |
|  | (3887 to 7652) | (17.44 to 31.79) | (8508 to 15925) | (17.39 to 31.61) | (1.02 to 1.24) | (-0.08 to 0.13) | (113 to 248) | (0.74 to 1.55) | (274 to 595) | (0.72 to 1.54) | (1.29 to 1.49) | (-0.15 to -0.01) |
| Kiribati | 10 | 13.94 | 17 | 13.99 | 0.62 | 0.04% | 0 | 0.68 | 1 | 0.67 | 0.73 | -0.03% |
|  | (8 to 14) | (10.22 to 18.72) | (12 to 23) | (10.36 to 18.78) | (0.52 to 0.75) | (-0.12 to 0.21) | (0 to 1) | (0.44 to 0.99) | (0 to 1) | (0.44 to 0.98) | (0.52 to 0.98) | (-0.19 to 0.13) |
| Kuwait | 505 | 26.64 | 1227 | 26.51 | 1.43 | -0.09% | 18 | 1.2 | 54 | 1.18 | 2.02 | -0.15% |
|  | (365 to 697) | (19.26 to 36.74) | (876 to 1732) | (18.97 to 37.26) | (1.29 to 1.57) | (-0.17 to -0.01) | (11 to 27) | (0.77 to 1.77) | (33 to 82) | (0.76 to 1.74) | (1.41 to 2.77) | (-0.22 to -0.08) |
| Kyrgyzstan | 1917 | 42.13 | 2256 | 33.52 | 0.18 | -0.90% | 72 | 1.88 | 91 | 1.5 | 0.26 | -0.85% |
|  | (1400 to 2640) | (30.78 to 57.73) | (1616 to 3141) | (24.08 to 46.62) | (0.1 to 0.26) | (-1.06 to -0.74) | (46 to 104) | (1.23 to 2.72) | (58 to 132) | (0.97 to 2.18) | (0.06 to 0.53) | (-0.97 to -0.74) |
| Latvia | 2330 | 86.5 | 1094 | 58.79 | -0.53 | -1.48% | 114 | 3.71 | 64 | 2.5 | -0.44 | -1.54% |
|  | (1664 to 3258) | (61.95 to 121.16) | (776 to 1557) | (42.18 to 82.75) | (-0.55 to -0.5) | (-1.6 to -1.35) | (74 to 162) | (2.44 to 5.35) | (42 to 92) | (1.64 to 3.59) | (-0.52 to -0.35) | (-1.66 to -1.42) |
| Lebanon | 987 | 29.89 | 1308 | 25.3 | 0.33 | -0.73% | 38 | 1.37 | 58 | 1.1 | 0.51 | -0.93% |
|  | (690 to 1391) | (20.8 to 41.99) | (931 to 1809) | (18.03 to 34.98) | (0.1 to 0.55) | (-1.12 to -0.35) | (25 to 56) | (0.88 to 1.98) | (37 to 86) | (0.7 to 1.62) | (0.23 to 0.84) | (-1.31 to -0.55) |
| Lesotho | 834 | 48.29 | 1196 | 58.06 | 0.43 | 0.77% | 30 | 2.22 | 46 | 2.63 | 0.52 | 0.73% |
|  | (611 to 1127) | (35.46 to 65.39) | (867 to 1632) | (42.1 to 80) | (0.31 to 0.56) | (0.68 to 0.86) | (19 to 45) | (1.43 to 3.24) | (30 to 67) | (1.75 to 3.83) | (0.32 to 0.75) | (0.68 to 0.78) |
| Liberia | 873 | 49.52 | 2012 | 48.58 | 1.3 | 0.02% | 33 | 2.36 | 73 | 2.25 | 1.22 | -0.09% |
|  | (623 to 1222) | (34.95 to 69.03) | (1429 to 2789) | (34.21 to 67.53) | (1.2 to 1.42) | (-0.09 to 0.12) | (22 to 47) | (1.57 to 3.38) | (47 to 105) | (1.48 to 3.28) | (0.94 to 1.53) | (-0.2 to 0.02) |
| Libya | 1080 | 25.73 | 2065 | 29.06 | 0.91 | 1.08% | 37 | 1.2 | 91 | 1.32 | 1.47 | 0.72% |
|  | (771 to 1483) | (18.55 to 35.6) | (1491 to 2802) | (21.07 to 39.48) | (0.7 to 1.21) | (0.39 to 1.79) | (24 to 54) | (0.78 to 1.73) | (59 to 132) | (0.87 to 1.91) | (1 to 2.05) | (0.38 to 1.07) |
| Lithuania | 2955 | 79.02 | 1740 | 61.94 | -0.41 | -0.79% | 138 | 3.38 | 100 | 2.63 | -0.28 | -0.84% |
|  | (2147 to 4143) | (57.25 to 110.61) | (1232 to 2481) | (44.46 to 88.14) | (-0.45 to -0.38) | (-0.9 to -0.69) | (91 to 198) | (2.24 to 4.86) | (66 to 145) | (1.73 to 3.83) | (-0.38 to -0.15) | (-0.94 to -0.74) |
| Luxembourg | 197 | 51.31 | 273 | 43.3 | 0.38 | -0.81% | 10 | 2.17 | 14 | 1.84 | 0.47 | -0.80% |
|  | (139 to 285) | (36.03 to 73.65) | (186 to 401) | (29.93 to 63.93) | (0.26 to 0.5) | (-0.88 to -0.73) | (6 to 14) | (1.41 to 3.1) | (9 to 21) | (1.16 to 2.75) | (0.24 to 0.73) | (-0.88 to -0.72) |
| Madagascar | 6898 | 63.41 | 14667 | 59.99 | 1.13 | -0.27% | 240 | 3.03 | 517 | 2.82 | 1.15 | -0.32% |
|  | (4944 to 9700) | (44.45 to 89.05) | (10446 to 20540) | (42.31 to 84.63) | (1.02 to 1.24) | (-0.35 to -0.18) | (155 to 346) | (2 to 4.34) | (333 to 760) | (1.86 to 4.06) | (0.88 to 1.48) | (-0.41 to -0.23) |
| Malawi | 4604 | 53.95 | 9195 | 54.62 | 1 | -0.10% | 156 | 2.53 | 310 | 2.5 | 0.99 | -0.16% |
|  | (3298 to 6442) | (38.35 to 76.34) | (6617 to 12805) | (39.12 to 75.71) | (0.89 to 1.12) | (-0.23 to 0.02) | (101 to 231) | (1.66 to 3.67) | (199 to 449) | (1.65 to 3.58) | (0.73 to 1.29) | (-0.26 to -0.07) |
| Malaysia | 3702 | 21.7 | 7074 | 22.48 | 0.91 | -0.05% | 138 | 1.01 | 303 | 1 | 1.19 | -0.16% |
|  | (2698 to 4906) | (15.96 to 28.8) | (5220 to 9470) | (16.5 to 30.11) | (0.79 to 1.05) | (-0.18 to 0.09) | (90 to 204) | (0.66 to 1.47) | (196 to 447) | (0.65 to 1.47) | (0.85 to 1.6) | (-0.28 to -0.03) |
| Maldives | 43 | 20.6 | 101 | 19.93 | 1.37 | -0.32% | 1 | 0.99 | 4 | 0.91 | 1.96 | -0.40% |
|  | (31 to 59) | (14.96 to 28.36) | (74 to 138) | (14.5 to 27.01) | (1.1 to 1.66) | (-1.34 to 0.72) | (1 to 2) | (0.64 to 1.45) | (3 to 6) | (0.6 to 1.31) | (1.48 to 2.58) | (-0.83 to 0.03) |
| Mali | 4089 | 54.34 | 9969 | 54.9 | 1.44 | 0.01% | 146 | 2.58 | 338 | 2.56 | 1.32 | -0.06% |
|  | (2936 to 5671) | (38.19 to 76.16) | (7164 to 13703) | (39.08 to 76.9) | (1.31 to 1.56) | (-0.06 to 0.08) | (93 to 211) | (1.69 to 3.71) | (223 to 488) | (1.72 to 3.64) | (1.04 to 1.64) | (-0.12 to 0.01) |
| Malta | 152 | 42.11 | 184 | 43.72 | 0.21 | 0.27% | 7 | 1.79 | 11 | 1.87 | 0.53 | 0.28% |
|  | (106 to 224) | (29.33 to 61.81) | (125 to 271) | (30.18 to 65.17) | (0.13 to 0.31) | (0.23 to 0.32) | (5 to 10) | (1.15 to 2.62) | (7 to 16) | (1.19 to 2.73) | (0.3 to 0.84) | (0.23 to 0.32) |
| Marshall Islands | 8 | 18.6 | 11 | 18.71 | 0.36 | -0.01% | 0 | 0.88 | 0 | 0.88 | 0.64 | -0.05% |
|  | (6 to 10) | (13.51 to 24.74) | (8 to 14) | (13.8 to 25.08) | (0.27 to 0.46) | (-0.07 to 0.06) | (0 to 0) | (0.59 to 1.3) | (0 to 1) | (0.58 to 1.29) | (0.4 to 0.94) | (-0.1 to 0) |
| Mauritania | 1141 | 63.78 | 2072 | 58.54 | 0.82 | -0.27% | 42 | 3.04 | 77 | 2.71 | 0.84 | -0.36% |
|  | (787 to 1621) | (43.11 to 92.09) | (1445 to 2952) | (40.13 to 83.36) | (0.74 to 0.92) | (-0.46 to -0.08) | (27 to 60) | (2.01 to 4.4) | (49 to 110) | (1.75 to 3.9) | (0.61 to 1.12) | (-0.56 to -0.16) |
| Mauritius | 556 | 50.59 | 806 | 60.62 | 0.45 | 0.64% | 22 | 2.26 | 41 | 2.64 | 0.86 | 0.58% |
|  | (395 to 775) | (35.9 to 70.72) | (563 to 1159) | (42.52 to 86.89) | (0.32 to 0.61) | (0.57 to 0.71) | (14 to 32) | (1.48 to 3.32) | (27 to 59) | (1.71 to 3.8) | (0.59 to 1.21) | (0.51 to 0.64) |
| Mexico | 86521 | 105.47 | 109732 | 87.62 | 0.27 | 1% | 3057 | 4.78 | 4744 | 3.82 | 0.55 | 0.88% |
|  | (59835 to 127295) | (72.85 to 156.26) | (75823 to 162316) | (60.3 to 129.54) | (0.21 to 0.33) | (0.53 to 1.48) | (1989 to 4427) | (3.18 to 6.83) | (3133 to 6771) | (2.52 to 5.46) | (0.47 to 0.64) | (0.4 to 1.37) |
| Mongolia | 912 | 41.3 | 1580 | 45.06 | 0.73 | 0.55% | 31 | 1.9 | 66 | 2.03 | 1.1 | 0.38% |
|  | (653 to 1292) | (29.32 to 58.75) | (1121 to 2256) | (31.96 to 63.93) | (0.61 to 0.87) | (0.46 to 0.63) | (20 to 47) | (1.22 to 2.82) | (42 to 98) | (1.31 to 2.99) | (0.76 to 1.49) | (0.31 to 0.44) |
| Montenegro | 457 | 72.38 | 423 | 70.23 | -0.08 | -0.14% | 20 | 3.12 | 23 | 3.02 | 0.14 | -0.15% |
|  | (316 to 659) | (50.15 to 104.43) | (290 to 619) | (48.72 to 102.88) | (-0.12 to -0.03) | (-0.2 to -0.07) | (13 to 29) | (2.02 to 4.62) | (15 to 33) | (2.01 to 4.34) | (-0.01 to 0.31) | (-0.22 to -0.09) |
| Morocco | 7051 | 26.98 | 9804 | 26.98 | 0.39 | -0.21% | 261 | 1.28 | 438 | 1.23 | 0.68 | -0.23% |
|  | (5023 to 9889) | (19.17 to 38.08) | (6940 to 13915) | (19.13 to 38.27) | (0.27 to 0.51) | (-0.41 to 0) | (167 to 394) | (0.83 to 1.91) | (291 to 647) | (0.82 to 1.81) | (0.38 to 1.02) | (-0.3 to -0.17) |
| Mozambique | 7758 | 67.32 | 20116 | 79.84 | 1.59 | 0.50% | 277 | 3.2 | 651 | 3.58 | 1.35 | 0.38% |
|  | (5555 to 10831) | (47 to 95.33) | (14335 to 27513) | (55.71 to 111.23) | (1.43 to 1.85) | (0.35 to 0.64) | (179 to 401) | (2.11 to 4.6) | (420 to 955) | (2.36 to 5.17) | (1.07 to 1.67) | (0.25 to 0.51) |
| Myanmar | 10328 | 24.99 | 12071 | 22.2 | 0.17 | 0.26% | 390 | 1.18 | 630 | 1.2 | 0.62 | 0.49% |
|  | (7498 to 13962) | (18.19 to 34) | (8725 to 16666) | (16.04 to 30.56) | (0.06 to 0.29) | (-1.63 to 2.18) | (256 to 573) | (0.78 to 1.72) | (414 to 908) | (0.79 to 1.73) | (0.29 to 1.19) | (-0.5 to 1.49) |
| Namibia | 695 | 53.56 | 1273 | 55.29 | 0.83 | 0.01% | 25 | 2.51 | 48 | 2.48 | 0.9 | -0.18% |
|  | (504 to 955) | (38.77 to 73.74) | (911 to 1752) | (39.8 to 76.57) | (0.72 to 0.94) | (-0.14 to 0.15) | (16 to 37) | (1.64 to 3.63) | (31 to 69) | (1.64 to 3.53) | (0.65 to 1.19) | (-0.33 to -0.03) |
| Nepal | 5232 | 28.23 | 8865 | 29.23 | 0.69 | 0.34% | 195 | 1.37 | 379 | 1.41 | 0.94 | 0.12% |
|  | (3847 to 6974) | (20.67 to 37.59) | (6477 to 12017) | (21.31 to 39.36) | (0.6 to 0.79) | (-0.41 to 1.09) | (124 to 283) | (0.87 to 1.99) | (252 to 549) | (0.94 to 2.03) | (0.66 to 1.33) | (-0.18 to 0.41) |
| Netherlands | 5143 | 34.15 | 7058 | 37.04 | 0.37 | 0.05% | 249 | 1.46 | 370 | 1.57 | 0.49 | 0.02% |
|  | (3606 to 7361) | (23.81 to 49.29) | (4773 to 10413) | (25.74 to 54.58) | (0.23 to 0.54) | (-0.19 to 0.3) | (163 to 361) | (0.96 to 2.13) | (243 to 538) | (1.03 to 2.3) | (0.24 to 0.79) | (-0.22 to 0.26) |
| New Zealand | 3383 | 99.01 | 4218 | 96.58 | 0.25 | -0.10% | 153 | 4.27 | 224 | 4.1 | 0.46 | -0.16% |
|  | (2277 to 5248) | (66.37 to 153.15) | (2882 to 6320) | (65.72 to 145.13) | (0.17 to 0.35) | (-0.14 to -0.06) | (101 to 223) | (2.82 to 6.21) | (150 to 327) | (2.74 to 6.01) | (0.32 to 0.62) | (-0.2 to -0.12) |
| Nicaragua | 1159 | 27.59 | 1706 | 25.87 | 0.47 | -0.70% | 37 | 1.27 | 68 | 1.16 | 0.85 | -0.51% |
|  | (830 to 1605) | (19.87 to 38.44) | (1227 to 2370) | (18.65 to 35.77) | (0.38 to 0.57) | (-1.65 to 0.27) | (24 to 55) | (0.82 to 1.87) | (44 to 98) | (0.75 to 1.66) | (0.56 to 1.22) | (-0.91 to -0.11) |
| Niger | 3717 | 54.74 | 10109 | 54.26 | 1.72 | -0.04% | 123 | 2.63 | 327 | 2.54 | 1.65 | -0.13% |
|  | (2657 to 5163) | (39.05 to 77.04) | (7237 to 14003) | (38.64 to 76.04) | (1.61 to 1.83) | (-0.13 to 0.05) | (81 to 179) | (1.77 to 3.77) | (211 to 475) | (1.67 to 3.68) | (1.35 to 1.98) | (-0.23 to -0.04) |
| Nigeria | 42017 | 52.83 | 96182 | 52.48 | 1.29 | 0.07% | 1517 | 2.46 | 3337 | 2.42 | 1.2 | -0.01% |
|  | (30125 to 58392) | (37.38 to 73.77) | (68817 to 132586) | (37.15 to 72.78) | (1.2 to 1.36) | (-0.03 to 0.17) | (995 to 2201) | (1.65 to 3.52) | (2189 to 4832) | (1.63 to 3.45) | (1.12 to 1.29) | (-0.11 to 0.09) |
| Northern Mariana Islands | 32 | 72.83 | 31 | 71.9 | -0.05 | -0.15% | 1 | 3.26 | 1 | 3.1 | 0.32 | -0.31% |
|  | (23 to 46) | (51.28 to 103.87) | (21 to 45) | (49.66 to 105.87) | (-0.15 to 0.07) | (-0.27 to -0.02) | (1 to 2) | (2.17 to 4.71) | (1 to 2) | (2.06 to 4.59) | (0.11 to 0.61) | (-0.4 to -0.23) |
| Norway | 6178 | 128.44 | 7718 | 122.61 | 0.25 | -0.30% | 286 | 5.32 | 372 | 5.04 | 0.3 | -0.33% |
|  | (3981 to 9753) | (83.63 to 197.44) | (4842 to 12667) | (78.27 to 195.47) | (0.17 to 0.32) | (-0.36 to -0.24) | (189 to 409) | (3.49 to 7.64) | (245 to 529) | (3.28 to 7.23) | (0.24 to 0.36) | (-0.39 to -0.26) |
| Oman | 606 | 32.5 | 1476 | 30.96 | 1.44 | -0.24% | 20 | 1.46 | 56 | 1.35 | 1.76 | -0.37% |
|  | (438 to 837) | (23.51 to 45.37) | (1067 to 2076) | (22.46 to 43.04) | (1.21 to 1.69) | (-0.36 to -0.12) | (13 to 30) | (0.95 to 2.13) | (34 to 84) | (0.87 to 1.95) | (1.13 to 2.51) | (-0.43 to -0.32) |
| Pakistan | 22124 | 20.97 | 50467 | 23.22 | 1.28 | 0.32% | 813 | 1.01 | 1882 | 1.11 | 1.31 | 0.35% |
|  | (16233 to 29254) | (15.34 to 27.62) | (37141 to 66310) | (16.96 to 30.77) | (1.13 to 1.46) | (-0.14 to 0.79) | (529 to 1170) | (0.66 to 1.46) | (1258 to 2715) | (0.74 to 1.57) | (1.09 to 1.56) | (0.18 to 0.52) |
| Palestine | 517 | 23.93 | 1384 | 26.54 | 1.68 | 0.43% | 21 | 1.39 | 51 | 1.27 | 1.41 | -0.31% |
|  | (372 to 721) | (17.09 to 33.17) | (992 to 1888) | (19.1 to 36.07) | (1.49 to 1.94) | (0.13 to 0.73) | (13 to 34) | (0.88 to 2.2) | (33 to 74) | (0.85 to 1.87) | (0.75 to 2.12) | (-0.48 to -0.14) |
| Panama | 730 | 29.73 | 1215 | 29.23 | 0.66 | -0.10% | 28 | 1.34 | 54 | 1.28 | 0.94 | -0.16% |
|  | (533 to 992) | (21.62 to 40.22) | (872 to 1702) | (20.99 to 40.92) | (0.57 to 0.79) | (-0.17 to -0.02) | (18 to 41) | (0.87 to 1.99) | (35 to 78) | (0.84 to 1.86) | (0.62 to 1.36) | (-0.22 to -0.11) |
| Papua New Guinea | 698 | 18.16 | 2018 | 21.51 | 1.89 | -0.07% | 26 | 0.88 | 78 | 1.06 | 2.06 | 0.41% |
|  | (512 to 933) | (13.32 to 24.1) | (1483 to 2738) | (15.75 to 29.17) | (1.69 to 2.11) | (-1.24 to 1.11) | (17 to 37) | (0.58 to 1.27) | (51 to 114) | (0.7 to 1.51) | (1.56 to 2.66) | (-0.09 to 0.91) |
| Paraguay | 1145 | 27.76 | 1927 | 27.14 | 0.68 | -0.14% | 42 | 1.29 | 80 | 1.22 | 0.92 | -0.24% |
|  | (810 to 1611) | (19.83 to 38.47) | (1393 to 2668) | (19.6 to 37.52) | (0.61 to 0.77) | (-0.21 to -0.06) | (27 to 61) | (0.85 to 1.88) | (52 to 117) | (0.8 to 1.78) | (0.62 to 1.31) | (-0.31 to -0.17) |
| Peru | 6644 | 29.07 | 8984 | 26.34 | 0.35 | -0.26% | 237 | 1.32 | 400 | 1.18 | 0.69 | -0.37% |
|  | (4865 to 9193) | (21.15 to 40.34) | (6439 to 12704) | (18.85 to 37.19) | (0.17 to 0.52) | (-0.37 to -0.15) | (152 to 349) | (0.85 to 1.92) | (259 to 592) | (0.77 to 1.73) | (0.42 to 1.03) | (-0.44 to -0.31) |
| Philippines | 17425 | 28.13 | 25414 | 22.74 | 0.46 | -0.43% | 567 | 1.18 | 1062 | 1.07 | 0.87 | -0.34% |
|  | (12525 to 23834) | (20.32 to 38.39) | (18307 to 34515) | (16.4 to 30.84) | (0.17 to 0.66) | (-0.98 to 0.12) | (374 to 832) | (0.79 to 1.7) | (717 to 1533) | (0.74 to 1.54) | (0.76 to 1) | (-0.53 to -0.15) |
| Poland | 30361 | 79.5 | 27009 | 71.1 | -0.11 | -0.42% | 1416 | 3.48 | 1517 | 3.05 | 0.07 | -0.49% |
|  | (21126 to 43903) | (55.16 to 114.73) | (18517 to 39559) | (49.14 to 103.3) | (-0.18 to -0.03) | (-0.51 to -0.32) | (942 to 2029) | (2.32 to 5.01) | (1013 to 2159) | (2.03 to 4.35) | (0.01 to 0.14) | (-0.6 to -0.39) |
| Portugal | 4056 | 40.27 | 3143 | 29.38 | -0.23 | -1.46% | 199 | 1.75 | 188 | 1.26 | -0.06 | -1.45% |
|  | (2857 to 5701) | (28.45 to 56.84) | (2190 to 4535) | (20.56 to 42.02) | (-0.28 to -0.16) | (-1.58 to -1.34) | (132 to 288) | (1.15 to 2.55) | (124 to 275) | (0.81 to 1.86) | (-0.21 to 0.12) | (-1.56 to -1.34) |
| Puerto Rico | 2760 | 75.98 | 3005 | 80.72 | 0.09 | 0.55% | 121 | 3.35 | 169 | 3.5 | 0.4 | 0.25% |
|  | (1981 to 3882) | (54.78 to 106.59) | (2101 to 4452) | (56.81 to 117.79) | (-0.04 to 0.23) | (0.16 to 0.93) | (80 to 176) | (2.2 to 4.87) | (113 to 242) | (2.31 to 5.1) | (0.21 to 0.62) | (0.11 to 0.38) |
| Qatar | 189 | 37.47 | 1186 | 35.08 | 5.29 | -0.09% | 7 | 1.7 | 45 | 1.53 | 5.63 | -0.29% |
|  | (134 to 268) | (26.79 to 52.49) | (840 to 1716) | (25.07 to 49.87) | (4.76 to 5.89) | (-0.19 to 0.01) | (4 to 10) | (1.11 to 2.51) | (28 to 69) | (0.98 to 2.26) | (3.95 to 7.96) | (-0.37 to -0.21) |
| Romania | 19252 | 82.53 | 12757 | 69.52 | -0.34 | -0.71% | 929 | 3.66 | 747 | 2.99 | -0.2 | -0.82% |
|  | (13163 to 28483) | (56.53 to 121.15) | (8642 to 19105) | (47.6 to 104.57) | (-0.37 to -0.3) | (-0.81 to -0.61) | (612 to 1357) | (2.41 to 5.34) | (490 to 1070) | (1.93 to 4.3) | (-0.3 to -0.05) | (-0.93 to -0.71) |
| Russian Federation | 123459 | 81.33 | 94606 | 64.42 | -0.23 | -0.79% | 5788 | 3.5 | 5071 | 2.77 | -0.12 | -0.83% |
|  | (87464 to 172970) | (57.87 to 114.04) | (67381 to 131702) | (46.07 to 89.17) | (-0.28 to -0.18) | (-0.99 to -0.58) | (3897 to 8278) | (2.35 to 5.02) | (3440 to 7250) | (1.86 to 3.98) | (-0.17 to -0.07) | (-1.04 to -0.61) |
| Rwanda | 4579 | 74.44 | 7295 | 63.25 | 0.59 | -0.76% | 158 | 3.55 | 262 | 2.88 | 0.66 | -0.89% |
|  | (3201 to 6462) | (50.78 to 107.26) | (5134 to 10277) | (43.81 to 89.48) | (0.49 to 0.72) | (-0.92 to -0.59) | (102 to 230) | (2.35 to 5.07) | (168 to 381) | (1.92 to 4.15) | (0.44 to 0.91) | (-1.01 to -0.78) |
| Saint Kitts and Nevis | 29 | 72.23 | 46 | 78.22 | 0.59 | 0.15% | 1 | 3.19 | 2 | 3.39 | 0.88 | 0.14% |
|  | (20 to 42) | (50.37 to 105.01) | (32 to 67) | (54.8 to 112.15) | (0.48 to 0.71) | (0.03 to 0.28) | (1 to 2) | (2.09 to 4.69) | (1 to 3) | (2.23 to 4.91) | (0.64 to 1.14) | (0.08 to 0.2) |
| Saint Lucia | 35 | 24.83 | 48 | 28 | 0.36 | 0.46% | 1 | 1.14 | 2 | 1.26 | 0.9 | 0.38% |
|  | (26 to 50) | (18.05 to 35.44) | (34 to 68) | (20.19 to 39.57) | (0.25 to 0.48) | (0.23 to 0.7) | (1 to 2) | (0.74 to 1.64) | (2 to 4) | (0.82 to 1.86) | (0.6 to 1.3) | (0.3 to 0.45) |
| Saint Vincent and the Grenadines | 29 | 25.91 | 32 | 28.9 | 0.1 | 0.40% | 1 | 1.18 | 2 | 1.33 | 0.52 | 0.42% |
|  | (22 to 41) | (18.96 to 36.4) | (23 to 46) | (20.78 to 41.03) | (0 to 0.2) | (0.08 to 0.71) | (1 to 2) | (0.76 to 1.71) | (1 to 2) | (0.87 to 1.96) | (0.25 to 0.83) | (0.33 to 0.51) |
| Samoa | 36 | 22.71 | 40 | 19.43 | 0.11 | 0.21% | 1 | 0.92 | 2 | 1.01 | 0.58 | 0.71% |
|  | (27 to 49) | (16.83 to 30.42) | (29 to 54) | (14.22 to 26.17) | (-0.13 to 0.29) | (-1.44 to 1.88) | (1 to 2) | (0.61 to 1.34) | (1 to 3) | (0.67 to 1.47) | (0.33 to 0.92) | (-0.09 to 1.51) |
| Sao Tome and Principe | 65 | 58.62 | 128 | 69.21 | 0.96 | 0.61% | 2 | 2.73 | 5 | 3.13 | 1.04 | 0.51% |
|  | (47 to 91) | (42.16 to 81.98) | (91 to 179) | (49.04 to 97.78) | (0.83 to 1.08) | (0.56 to 0.66) | (2 to 3) | (1.84 to 3.91) | (3 to 7) | (2.07 to 4.51) | (0.8 to 1.35) | (0.46 to 0.56) |
| Saudi Arabia | 7593 | 47.42 | 22476 | 55.53 | 1.96 | 0.66% | 255 | 2.18 | 864 | 2.43 | 2.39 | 0.48% |
|  | (5297 to 10825) | (33.24 to 67.32) | (15643 to 32839) | (38.87 to 81.04) | (1.5 to 2.39) | (0.54 to 0.77) | (163 to 382) | (1.43 to 3.18) | (552 to 1293) | (1.55 to 3.53) | (1.77 to 3.22) | (0.37 to 0.58) |
| Senegal | 3325 | 51.3 | 6907 | 52.94 | 1.08 | 0.14% | 116 | 2.43 | 254 | 2.45 | 1.2 | 0.07% |
|  | (2354 to 4732) | (35.68 to 72.69) | (4888 to 9669) | (36.99 to 75.61) | (0.98 to 1.17) | (0.07 to 0.2) | (75 to 167) | (1.6 to 3.48) | (163 to 368) | (1.62 to 3.52) | (0.93 to 1.49) | (0 to 0.14) |
| Serbia | 6123 | 66.33 | 5460 | 65.68 | -0.11 | -0.03% | 306 | 2.96 | 307 | 2.82 | 0 | -0.18% |
|  | (4201 to 9079) | (45.72 to 97.82) | (3732 to 8247) | (44.83 to 98.68) | (-0.17 to -0.04) | (-0.12 to 0.07) | (197 to 450) | (1.92 to 4.36) | (203 to 453) | (1.85 to 4.15) | (-0.15 to 0.18) | (-0.27 to -0.08) |
| Seychelles | 42 | 58.91 | 64 | 59.95 | 0.53 | -0.10% | 2 | 2.65 | 3 | 2.63 | 0.76 | -0.17% |
|  | (29 to 58) | (41.87 to 82.68) | (45 to 90) | (42.27 to 83.91) | (0.42 to 0.63) | (-0.23 to 0.02) | (1 to 2) | (1.73 to 3.82) | (2 to 4) | (1.74 to 3.87) | (0.49 to 1.06) | (-0.28 to -0.07) |
| Sierra Leone | 1726 | 52.71 | 3816 | 54.06 | 1.21 | 0.34% | 64 | 2.52 | 139 | 2.56 | 1.16 | 0.18% |
|  | (1221 to 2412) | (36.38 to 74.88) | (2682 to 5307) | (37.75 to 76.83) | (1.11 to 1.32) | (0.02 to 0.66) | (42 to 93) | (1.68 to 3.67) | (92 to 201) | (1.71 to 3.69) | (0.89 to 1.47) | (0.05 to 0.3) |
| Singapore | 1463 | 44.81 | 2235 | 40.31 | 0.53 | -0.53% | 60 | 1.97 | 124 | 1.78 | 1.08 | -0.49% |
|  | (1006 to 2161) | (30.95 to 65.83) | (1546 to 3330) | (27.85 to 59.82) | (0.44 to 0.63) | (-0.63 to -0.44) | (38 to 87) | (1.29 to 2.85) | (80 to 181) | (1.16 to 2.6) | (0.71 to 1.54) | (-0.58 to -0.41) |
| Slovakia | 4479 | 84.03 | 4243 | 77.79 | -0.05 | -0.30% | 202 | 3.61 | 229 | 3.3 | 0.13 | -0.34% |
|  | (3060 to 6609) | (57.56 to 123.88) | (2877 to 6272) | (53.03 to 114.99) | (-0.11 to 0.01) | (-0.37 to -0.23) | (134 to 289) | (2.39 to 5.17) | (147 to 335) | (2.12 to 4.81) | (-0.01 to 0.3) | (-0.43 to -0.26) |
| Slovenia | 1929 | 95.57 | 1787 | 84.14 | -0.07 | -0.03% | 89 | 4.01 | 102 | 3.58 | 0.15 | 0.03% |
|  | (1313 to 2869) | (65.27 to 140.85) | (1210 to 2661) | (57.15 to 125.63) | (-0.14 to 0) | (-0.21 to 0.15) | (58 to 128) | (2.65 to 5.79) | (67 to 147) | (2.35 to 5.27) | (0.01 to 0.32) | (-0.14 to 0.2) |
| Solomon Islands | 68 | 22.2 | 142 | 23.04 | 1.08 | 0.31% | 3 | 1.12 | 5 | 1.11 | 1.14 | -0.01% |
|  | (50 to 93) | (16.27 to 30.11) | (102 to 199) | (16.46 to 32.11) | (0.91 to 1.27) | (-0.16 to 0.79) | (2 to 4) | (0.76 to 1.59) | (4 to 8) | (0.74 to 1.59) | (0.79 to 1.55) | (-0.15 to 0.13) |
| Somalia | 3817 | 62.68 | 11296 | 64.45 | 1.96 | 0.24% | 129 | 2.98 | 374 | 3.02 | 1.89 | 0.09% |
|  | (2782 to 5311) | (45.2 to 87.35) | (8212 to 15506) | (46.53 to 89.39) | (1.85 to 2.09) | (0.02 to 0.46) | (83 to 191) | (1.98 to 4.28) | (243 to 554) | (2 to 4.37) | (1.51 to 2.34) | (0.01 to 0.17) |
| South Africa | 23296 | 65.42 | 29383 | 52.69 | 0.26 | -0.50% | 891 | 3.07 | 1251 | 2.38 | 0.4 | -0.62% |
|  | (16571 to 31749) | (46.11 to 89.49) | (21112 to 40182) | (37.75 to 71.88) | (0.18 to 0.33) | (-0.71 to -0.29) | (591 to 1283) | (2.05 to 4.35) | (840 to 1778) | (1.61 to 3.38) | (0.31 to 0.51) | (-0.84 to -0.39) |
| South Sudan | 3447 | 67.78 | 5253 | 64.29 | 0.52 | 0.16% | 120 | 3.22 | 187 | 3.08 | 0.56 | -0.08% |
|  | (2451 to 4864) | (47.52 to 94.49) | (3803 to 7310) | (45.58 to 89.27) | (0.46 to 0.6) | (-0.11 to 0.43) | (78 to 173) | (2.14 to 4.61) | (124 to 270) | (2.05 to 4.42) | (0.37 to 0.79) | (-0.19 to 0.03) |
| Spain | 15707 | 40.65 | 16555 | 37.02 | 0.05 | -0.52% | 769 | 1.75 | 990 | 1.61 | 0.29 | -0.50% |
|  | (11080 to 22463) | (28.72 to 57.82) | (11292 to 24653) | (25.4 to 55.6) | (-0.05 to 0.18) | (-0.59 to -0.46) | (494 to 1126) | (1.11 to 2.57) | (645 to 1434) | (1.03 to 2.36) | (0.06 to 0.57) | (-0.57 to -0.43) |
| Sri Lanka | 4582 | 26.83 | 5431 | 24.46 | 0.19 | -0.53% | 180 | 1.24 | 273 | 1.15 | 0.51 | -0.35% |
|  | (3319 to 6202) | (19.5 to 36.22) | (3924 to 7495) | (17.66 to 33.6) | (0.05 to 0.35) | (-1.95 to 0.91) | (115 to 266) | (0.8 to 1.81) | (178 to 398) | (0.75 to 1.67) | (0.22 to 0.89) | (-1.06 to 0.36) |
| Sudan | 11093 | 57.05 | 24414 | 60.18 | 1.2 | 0.10% | 390 | 2.73 | 848 | 2.75 | 1.18 | -0.04% |
|  | (7876 to 15790) | (40.4 to 80.6) | (17351 to 34944) | (42.84 to 85.56) | (1.01 to 1.4) | (-0.03 to 0.24) | (252 to 575) | (1.79 to 3.97) | (541 to 1245) | (1.79 to 3.96) | (0.87 to 1.56) | (-0.1 to 0.02) |
| Suriname | 94 | 23.34 | 148 | 26.14 | 0.59 | 0.43% | 4 | 1.18 | 7 | 1.22 | 0.79 | 0.14% |
|  | (69 to 130) | (17.3 to 32.57) | (109 to 208) | (19.26 to 36.58) | (0.5 to 0.68) | (0.4 to 0.45) | (3 to 6) | (0.78 to 1.79) | (5 to 11) | (0.81 to 1.79) | (0.42 to 1.23) | (0.11 to 0.17) |
| Sweden | 11851 | 124.55 | 14959 | 124.61 | 0.26 | -0.06% | 589 | 5.24 | 761 | 5.21 | 0.29 | -0.09% |
|  | (7692 to 18572) | (81.66 to 193.42) | (9435 to 24195) | (79.47 to 196.79) | (0.17 to 0.34) | (-0.15 to 0.04) | (379 to 851) | (3.37 to 7.62) | (499 to 1085) | (3.36 to 7.56) | (0.18 to 0.41) | (-0.2 to 0.01) |
| Switzerland | 4410 | 61.01 | 4537 | 48.84 | 0.03 | -1.20% | 213 | 2.55 | 247 | 2.07 | 0.16 | -1.17% |
|  | (2962 to 6510) | (41.1 to 90.29) | (3066 to 6825) | (33.21 to 73.78) | (-0.05 to 0.1) | (-1.38 to -1.01) | (139 to 308) | (1.65 to 3.67) | (165 to 356) | (1.36 to 2.99) | (-0.02 to 0.39) | (-1.37 to -0.98) |
| Tajikistan | 2172 | 39.61 | 3237 | 32.48 | 0.49 | -0.93% | 74 | 1.79 | 124 | 1.51 | 0.68 | -0.75% |
|  | (1566 to 3001) | (28.53 to 54.62) | (2287 to 4595) | (22.93 to 46.43) | (0.38 to 0.6) | (-1.31 to -0.54) | (48 to 108) | (1.16 to 2.65) | (80 to 183) | (0.98 to 2.22) | (0.42 to 0.94) | (-0.97 to -0.54) |
| Thailand | 15684 | 27.17 | 16621 | 22.7 | 0.06 | -0.83% | 617 | 1.25 | 889 | 1.01 | 0.44 | -0.93% |
|  | (11450 to 21009) | (19.77 to 36.21) | (12289 to 22423) | (16.75 to 30.73) | (-0.05 to 0.19) | (-1.1 to -0.57) | (394 to 906) | (0.81 to 1.82) | (582 to 1294) | (0.66 to 1.47) | (0.19 to 0.74) | (-1.03 to -0.83) |
| Timor-Leste | 145 | 19 | 241 | 18.61 | 0.66 | -0.14% | 5 | 0.92 | 9 | 0.87 | 0.8 | -0.29% |
|  | (106 to 198) | (13.8 to 25.82) | (175 to 327) | (13.56 to 25.03) | (0.55 to 0.8) | (-0.26 to -0.03) | (3 to 8) | (0.61 to 1.36) | (6 to 13) | (0.58 to 1.27) | (0.57 to 1.06) | (-0.4 to -0.17) |
| Togo | 1779 | 56.55 | 4049 | 57.59 | 1.28 | 0.10% | 58 | 2.66 | 147 | 2.65 | 1.52 | 0.01% |
|  | (1253 to 2517) | (39.53 to 80.32) | (2845 to 5707) | (40.28 to 81.09) | (1.17 to 1.39) | (0.02 to 0.19) | (38 to 85) | (1.74 to 3.83) | (98 to 217) | (1.78 to 3.85) | (1.24 to 1.88) | (-0.05 to 0.06) |
| Tokelau | 1 | 48.82 | 1 | 54.31 | -0.03 | 0.37% | 0 | 2.27 | 0 | 2.44 | 0.03 | 0.23% |
|  | (1 to 1) | (34.39 to 67.58) | (1 to 1) | (38.11 to 78.15) | (-0.1 to 0.07) | (0.3 to 0.45) | (0 to 0) | (1.48 to 3.3) | (0 to 0) | (1.56 to 3.59) | (-0.11 to 0.21) | (0.15 to 0.32) |
| Tonga | 17 | 18.31 | 16 | 15.96 | -0.05 | -0.33% | 1 | 0.85 | 1 | 0.76 | 0.1 | -0.39% |
|  | (12 to 23) | (13.3 to 24.66) | (12 to 22) | (11.57 to 21.69) | (-0.15 to 0.06) | (-0.83 to 0.17) | (0 to 1) | (0.56 to 1.23) | (0 to 1) | (0.5 to 1.08) | (-0.05 to 0.26) | (-0.63 to -0.15) |
| Trinidad and Tobago | 279 | 22.61 | 334 | 25.16 | 0.2 | 0.56% | 11 | 1.03 | 18 | 1.13 | 0.61 | 0.49% |
|  | (207 to 384) | (16.76 to 31.13) | (245 to 467) | (18.51 to 35.18) | (0.11 to 0.29) | (0.47 to 0.66) | (7 to 16) | (0.66 to 1.51) | (12 to 27) | (0.73 to 1.67) | (0.31 to 0.97) | (0.41 to 0.57) |
| Tunisia | 2272 | 26.66 | 3003 | 25.88 | 0.32 | 0.01% | 84 | 1.21 | 144 | 1.16 | 0.72 | -0.21% |
|  | (1600 to 3238) | (18.76 to 38.23) | (2097 to 4296) | (18.13 to 36.9) | (0.21 to 0.44) | (-0.1 to 0.12) | (54 to 123) | (0.79 to 1.77) | (94 to 214) | (0.75 to 1.71) | (0.43 to 1.08) | (-0.26 to -0.15) |
| Turkmenistan | 1342 | 34.16 | 1858 | 35.61 | 0.38 | 0.24% | 47 | 1.56 | 78 | 1.59 | 0.67 | 0.14% |
|  | (965 to 1878) | (24.47 to 47.97) | (1297 to 2700) | (24.82 to 51.91) | (0.28 to 0.5) | (0.13 to 0.35) | (30 to 68) | (1 to 2.28) | (50 to 115) | (1.03 to 2.34) | (0.39 to 1.02) | (0.04 to 0.24) |
| Tuvalu | 4 | 48.06 | 6 | 54.17 | 0.48 | 0.37% | 0 | 2.25 | 0 | 2.46 | 0.52 | 0.29% |
|  | (3 to 6) | (33.84 to 66.79) | (4 to 9) | (38.25 to 77.25) | (0.38 to 0.61) | (0.3 to 0.44) | (0 to 0) | (1.47 to 3.26) | (0 to 0) | (1.61 to 3.51) | (0.3 to 0.8) | (0.21 to 0.36) |
| Uganda | 8734 | 57.04 | 21522 | 58.54 | 1.46 | 0.03% | 286 | 2.67 | 694 | 2.68 | 1.43 | -0.06% |
|  | (6265 to 12090) | (40.35 to 80.71) | (15367 to 29995) | (41.24 to 82.45) | (1.36 to 1.57) | (-0.04 to 0.1) | (185 to 417) | (1.76 to 3.89) | (445 to 1010) | (1.76 to 3.84) | (1.11 to 1.78) | (-0.13 to 0) |
| Ukraine | 38944 | 74.67 | 27463 | 64.78 | -0.29 | -0.78% | 1939 | 3.23 | 1544 | 2.77 | -0.2 | -0.85% |
|  | (27642 to 55335) | (53.27 to 106.23) | (19418 to 38816) | (45.87 to 91.76) | (-0.32 to -0.27) | (-0.96 to -0.6) | (1277 to 2745) | (2.14 to 4.61) | (1009 to 2226) | (1.79 to 4.02) | (-0.32 to -0.06) | (-1.03 to -0.67) |
| United Arab Emirates | 599 | 30.38 | 2994 | 29.08 | 4 | -0.24% | 22 | 1.4 | 138 | 1.33 | 5.37 | -0.29% |
|  | (430 to 831) | (21.9 to 41.91) | (2171 to 4223) | (21.1 to 40.13) | (3.61 to 4.4) | (-0.29 to -0.18) | (14 to 32) | (0.92 to 2.06) | (80 to 219) | (0.84 to 2) | (3.49 to 8.04) | (-0.34 to -0.24) |
| United Kingdom | 28712 | 50.82 | 34737 | 50.02 | 0.21 | -0.08% | 1449 | 2.16 | 1862 | 2.14 | 0.29 | -0.06% |
|  | (20058 to 41522) | (35.57 to 73.75) | (23504 to 52021) | (33.97 to 74.94) | (0.12 to 0.31) | (-0.15 to -0.01) | (954 to 2101) | (1.42 to 3.12) | (1237 to 2676) | (1.4 to 3.09) | (0.23 to 0.34) | (-0.13 to 0.01) |
| Uruguay | 1973 | 63.47 | 1851 | 54.64 | -0.06 | -0.74% | 96 | 2.86 | 96 | 2.39 | 0 | -0.85% |
|  | (1260 to 3248) | (40.48 to 105.36) | (1247 to 2850) | (36.68 to 84.22) | (-0.15 to 0.05) | (-0.84 to -0.63) | (62 to 142) | (1.83 to 4.26) | (63 to 143) | (1.54 to 3.56) | (-0.14 to 0.17) | (-0.96 to -0.74) |
| Uzbekistan | 8144 | 37.15 | 12478 | 35.58 | 0.53 | -0.24% | 285 | 1.69 | 494 | 1.59 | 0.73 | -0.30% |
|  | (5834 to 11440) | (26.53 to 51.76) | (8982 to 17486) | (25.55 to 49.81) | (0.44 to 0.62) | (-0.3 to -0.18) | (186 to 418) | (1.11 to 2.49) | (314 to 732) | (1.03 to 2.34) | (0.47 to 1.04) | (-0.36 to -0.24) |
| Vanuatu | 23 | 16.16 | 48 | 16.57 | 1.04 | -0.70% | 1 | 0.86 | 2 | 0.89 | 1.26 | -0.21% |
|  | (17 to 31) | (11.81 to 21.56) | (35 to 65) | (12.2 to 22.44) | (0.92 to 1.18) | (-2.25 to 0.87) | (1 to 1) | (0.57 to 1.2) | (1 to 3) | (0.6 to 1.26) | (0.95 to 1.59) | (-0.88 to 0.46) |
| Yemen | 3229 | 24 | 7297 | 22.79 | 1.26 | -0.17% | 109 | 1.19 | 258 | 1.09 | 1.37 | -0.31% |
|  | (2321 to 4507) | (17.11 to 33.15) | (5236 to 9990) | (16.45 to 31.48) | (1.09 to 1.43) | (-0.33 to -0.01) | (72 to 158) | (0.79 to 1.73) | (163 to 380) | (0.71 to 1.58) | (1.01 to 1.83) | (-0.36 to -0.25) |
| Zambia | 3778 | 55.18 | 9100 | 56.82 | 1.41 | 0.06% | 125 | 2.62 | 305 | 2.58 | 1.43 | -0.10% |
|  | (2743 to 5262) | (40.06 to 77.24) | (6506 to 12697) | (40.68 to 78.09) | (1.28 to 1.55) | (-0.01 to 0.12) | (82 to 183) | (1.75 to 3.79) | (193 to 452) | (1.7 to 3.73) | (1.1 to 1.79) | (-0.16 to -0.05) |
| Zimbabwe | 4313 | 46.81 | 7220 | 51.78 | 0.67 | -0.09% | 143 | 2.15 | 234 | 2.14 | 0.63 | -0.19% |
|  | (3103 to 5961) | (34.02 to 64.26) | (5290 to 9663) | (37.89 to 68.82) | (0.51 to 1.06) | (-0.27 to 0.09) | (93 to 207) | (1.39 to 3.08) | (154 to 341) | (1.42 to 3.07) | (0.41 to 0.89) | (-0.31 to -0.07) |
